# Low-viscosity matrix suspension culture enables scalable analysis of patient-derived organoids and tumoroids from the large intestine

**DOI:** 10.1038/s42003-021-02607-y

**Published:** 2021-09-13

**Authors:** Yumiko Hirokawa, Jordan Clarke, Michelle Palmieri, Tao Tan, Dmitri Mouradov, Shan Li, Cong Lin, Fuqiang Li, Huijuan Luo, Kui Wu, Maree Faux, Chin Wee Tan, Margaret Lee, Grace Gard, Peter Gibbs, Antony W. Burgess, Oliver M. Sieber

**Affiliations:** 1 Personalised Oncology Division, The Walter and Eliza Hall Institute of Medial Research, Parkville, Victoria 3052 Australia; 2 Department of Medical Biology, The University of Melbourne, Parkville, Victoria 3052 Australia; 3 BGI-Shenzhen, Shenzhen, 518083 China; 4 Guangdong Provincial Key Laboratory of Human Disease Genomics, Shenzhen Key Laboratory of Genomics, BGI-Shenzhen, Shenzhen, 518083 China; 5 Department of Medical Oncology, Western Health, Footscray, Victoria 3011 Australia; 6 Department of Medical Oncology, Eastern Health, Box Hill, Victoria, 3128 Australia; 7 Department of Surgery, The University of Melbourne, Parkville, Victoria 3050 Australia; 8 Department of Biochemistry and Molecular Biology, Monash University, Clayton, Victoria 3800 Australia

**Keywords:** Cancer models, Colorectal cancer

## Abstract

Cell embedment into a solid support matrix is considered essential for the culture of intestinal epithelial organoids and tumoroids, but this technique presents challenges that impede scalable culture expansion, experimental manipulation, high-throughput screening and diagnostic applications. We have developed a low-viscosity matrix (LVM) suspension culture method that enables efficient establishment and propagation of organoids and tumoroids from the human large intestine. Organoids and tumoroids cultured in LVM suspension recapitulate the morphological development observed in solid matrices, with tumoroids reflecting the histological features and genetic heterogeneity of primary colorectal cancers. We demonstrate the utility of LVM suspension culture for organoid and tumoroid bioreactor applications and biobanking, as well as tumoroid high-throughput drug sensitivity testing. These methods provide opportunities for the study and use of patient-derived organoids and tumoroids from the large intestine.

## Abstract (web summary)

Given the practical limitations of solid matrix-based protocols in organoid culture, Yumiko Hirokawa et al. assess the ability of low-concentration Matrigel conditions to promote intestinal organoid growth. Their results suggest that a low-viscosity culture system can improve live cell yield compared to the existing dome method, while maintaining similar morphology, and represents a useful approach for high-throughput applications of organoids.

## Introduction

Organoids and tumoroids are self-organizing three-dimensional (3D) in vitro tissue models that recapitulate many of the physiologically relevant features of the normal or neoplastic tissue from which they are derived. Organoids and tumoroids from the human large intestine can be established from primary stem cells of intestinal crypts and tumor fragments, respectively^1–3^. When embedded into a solid support matrix, such as Matrigel or a synthetic hydrogel, epithelial stem cells avoid anoikis, proliferate, differentiate, and self-renew^2,4^. Organoid cultures of normal colorectal epithelium are grown in media supplemented with growth factors and inhibitors mimicking the niche environment necessary for the renewal of intestinal stem cells^5,6^. This includes stimulation of Wnt and EGF signaling, and inhibition of BMP, TGF-β, and p38 signaling^1–3^. Cells grow into mature organoids comprising stem and differentiated cells, organized into a sphere-shaped lumen and crypt-like protrusions similar to normal colorectal epithelium^2,7^. These conditions also support the growth of human colorectal adenoma and carcinoma tumoroids, although tumor niche factor requirements are more adaptable, with Wnt ligands commonly omitted to enable selective outgrowth of Wnt pathway mutated tumor cells (~90% of cases)^8,9^. Mature organoids and tumoroids can be passaged and biobanked, maintaining genetic fidelity to their original tissue for an extended period^8,10–14^, and therefore can be used as experimental, diagnostic, and potentially therapeutic tools^13–18^.

While cell embedment into a solid support matrix is considered essential for intestinal epithelial organoid and tumoroid culture, this presents technical challenges that impede culture expansion, experimentation, and high-throughput screening applications. Organoid and tumoroid growth in solid matrices are constrained due to solid stress accumulation, oxygen, and nutrient delivery^19^, requiring frequent passaging to maintain and expand cultures. Solid matrices must be mechanically or enzymatically removed to isolate organoids and tumoroids for propagation or experimental applications^2^. The preparation and handling of solid matrices increases the complexity of organoid and tumoroid assay design and automation, involving additional liquid-handling steps and/or cooling requirements^8,13,15,20–22^.

We have developed a low-viscosity matrix suspension (LVM) culture method that enables the growth of organoids and tumoroids from the human large intestine. We demonstrate the utility of this suspension culture approach for organoid and tumoroid establishment, propagation, scalable expansion, and biobanking, as well as tumoroid high-throughput drug screening and diagnostic testing.

## Results

### Development of a LVM suspension culture method for propagation of organoids and tumoroids from the human large intestine

The use of a solid support matrix poses challenges to organoid and tumoroid passaging, expansion, and assay automation with implications for assay costs. To address these challenges, and based on our findings that intestinal organoids can establish when only partially touching a support matrix^23^, we examined whether the traditional solid matrix could be replaced with a low-viscosity (3–5% Matrigel) matrix suspension which, unlike high-percentage Matrigel matrix, does not solidify at 37 °C. Organoids were grown in commercially available IntestiCult Organoid Growth Medium, tailored to the niche requirements for growth of normal intestinal epithelium^5,6^, while tumoroids were grown in reduced medium (DMEM/F12, HEPES, B27 supplement, N2 supplement, nicotinamide, N-acetyl-L-cysteine, bFGF, EGF, penicillin-streptomycin, and normocin) lacking Wnt agonists, BMP, TGF-β, and p38 inhibitors^2,8,10^.

**Fig. 1:**
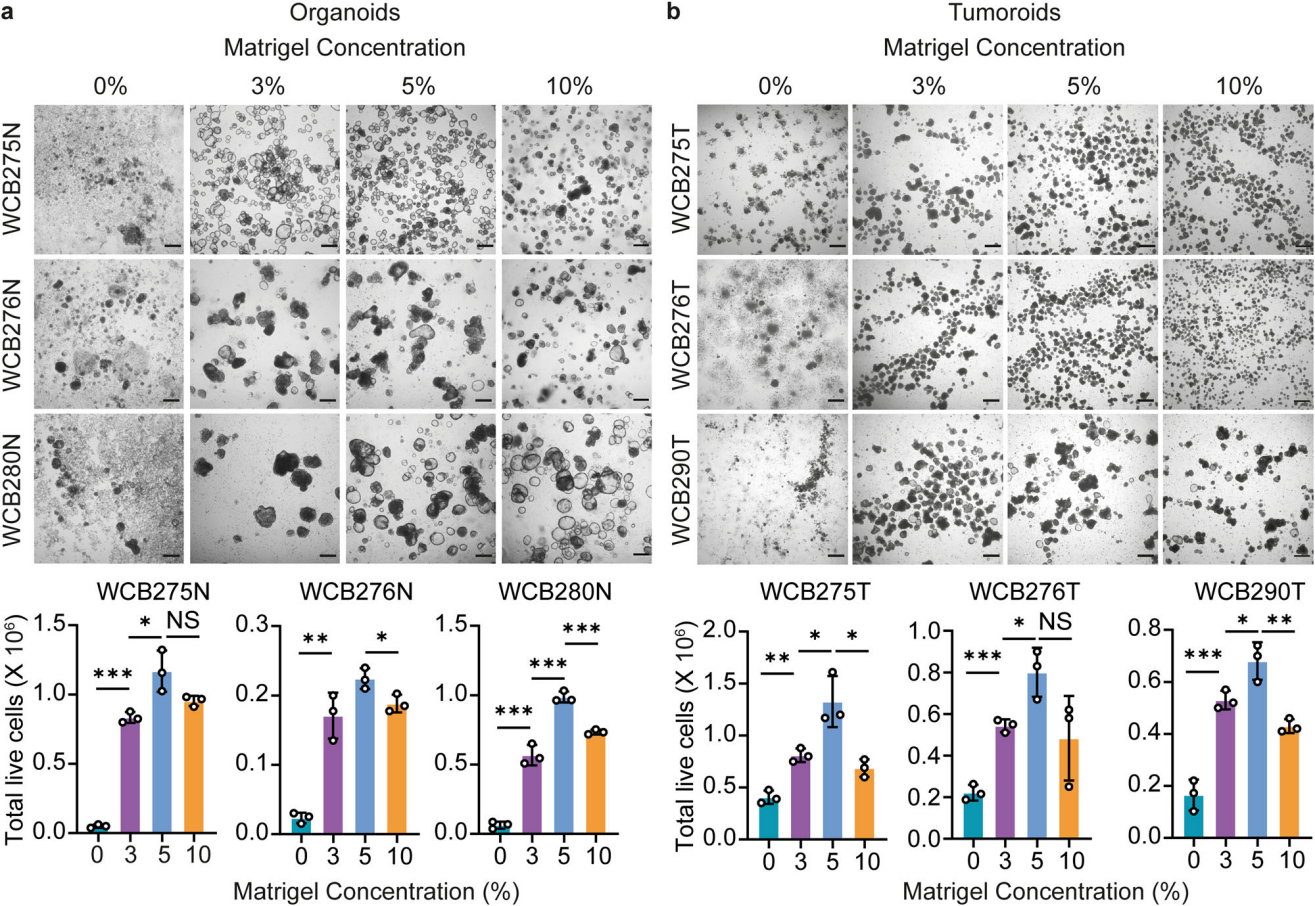
Low-percentage, low-viscosity Matrigel for suspension culture of patient-derived colorectal organoids and tumoroids. **a**–**b** Representative bright-field images and quantification of live-cell yield for (**a**) organoids and (**b**) tumoroids from three patients grown from single cells in media supplemented with 0, 3, 5, or 10% of Matrigel matrix (25,000 cells, 1 ml of medium/well) over a 14-day incubation period; scale bars, 200 μm. Data are plotted as mean ± s.d. Statistical significance was attributed to values of *p* < 0.05 as determined by the Student’s *t* test. NS, *p* > 0.05, *p < 0.05, **p < 0.01, ***p < 0.001. N normal, T tumor.

Single cells of already established (passage 1) organoid or tumoroid cultures from three patients were suspended in media supplemented with 0, 3, 5, or 10% of Matrigel matrix (25,000 cells, 1 ml of medium/well) and examined over a 14-day period (Fig. 1a, b). Cells suspended in medium without Matrigel tended to adhere to well bottoms and cease to grow, with only limited formation of organoids or tumoroids and the latter producing moderately larger numbers. In the presence of 3% Matrigel, cells efficiently formed organoids or tumoroids resulting in significantly higher live-cell yields (organoids: 9–16-fold increase; tumoroids: two to threefold increase); with increasing size, organoids or tumoroids tended to gravitate and adhere to well bottoms, requiring gentle agitation of cultures by pipetting every 2–3 days (Supplementary Fig. 1). Live-cell yields were further improved in 5% Matrigel as compared to 3% Matrigel for both organoids and tumoroids (organoids: 1.3–1.7-fold increase; tumoroids: 1.3–1.6-fold increase), whilst there was a decrease in yields between 5 and 10% Matrigel cultures. Based on these observations and considering the increase in media viscosity with higher Matrigel concentrations, we selected 5% Matrigel for further examination of the efficiency of LVM suspension culture.

**Fig. 2:**
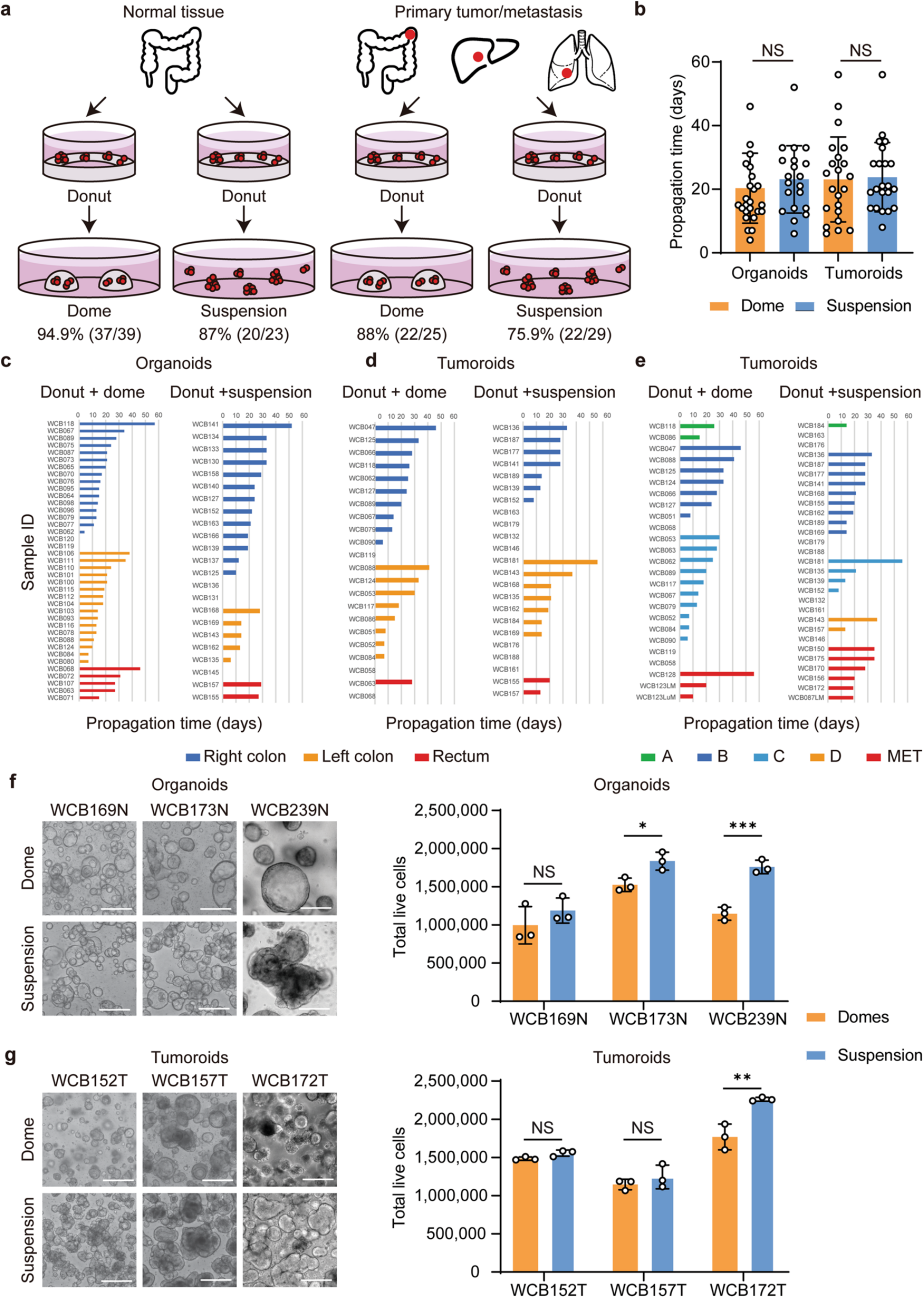
Propagation of colorectal organoids and tumoroids in low-viscosity matrix suspension culture. **a**–**b** Comparison of (**a**) propagation rates and (**b**) times (passage 1 to 2) between LVM suspension and dome culture methods for organoids (*n* = 23 and *n* = 39, respectively) and tumoroids (*n* = 29 and *n* = 25, respectively). **c**–**e** Comparison of propagation times for organoids and tumoroids grown in LVM suspension or dome culture according to (**c**, **d**) location and (**e**) tumor stage. **f**–**g** Comparison of live-cell yield for (**f**) organoids and (**g**) tumoroids from three patients grown for 14 days in LVM suspension or dome culture; scale bars, 200 μm. Data (**b**, **f**, **g**) are plotted as mean ± s.d. Statistical significance (**b**, **f**, **g**) was attributed to values of *p* < 0.05 as determined by the Student’s *t* test. NS, *p* > 0.05, *p < 0.05, **p < 0.01, ***p < 0.001. N normal, T tumor.

To compare propagation efficiencies of colorectal organoids and tumoroids (passage 1–2) between LVM suspension and Matrigel dome cultures, in which cells are grown embedded in a solidified drop of Matrigel, cultures were established for 62 normal colorectal and 54 cancer tissues. For organoids derived from normal colorectal epithelium, LVM suspension cultures achieved similar success as compared to dome cultures, with propagation rates of 87.0% (20/23) and 94.9% (37/39; *p* = 0.350), respectively (Fig. 2a, Supplementary Fig. 2a,b). Corresponding results were obtained for tumoroids, with propagation rates of 75.9% (22/29) in LVM suspension and 88.0% in dome culture (22/25; *p* = 0.310) (Fig. 2a, Supplementary Fig. 2c, d). There was no significant difference in propagation times between cultures grown using either method for both the organoids (LVM: mean = 23.1 days, s.d. = 10.6; dome: mean = 20.3 days, s.d. = 11.0; *p* = 0.362) and tumoroids (LVM: mean = 21.5 days, s.d. = 13.9; dome: mean = 24.2 days, s.d. = 13.0; *p* = 0.854) (Fig. 2b). Propagation rates and times were similar for organoids irrespective of the intestinal tract location (Fig. 2c, Supplementary Tables 1 and 2) and for tumoroids irrespective of location and tumor stage (Fig. 2d, e, Supplementary Tables 1 and 2). Organoids and tumoroids in LVM suspension culture could be readily biobanked and recovered with 100% (12/12) and 94.7% (18/19) success rates, respectively.

We further compared live-cell yields for organoids and tumoroids propagated in LVM suspension or dome culture over a 14-day period. Both the organoids and tumoroids from three patients with colorectal cancer were grown from single cells (100,000 cells, 3.5 ml of medium/well) either suspended in 5% of Matrigel matrix or embedded in solid Matrigel matrix with regular media changes every 2 days. Compared to dome cultures, both the organoids and tumoroids grown in LVM suspension culture tended to produce greater yields of viable cells (organoids: 1.2–1.5-fold increase; tumoroids: 1.0–1.3-fold increase) (Fig. 2f–g).

Besides Matrigel matrix, other commonly used support matrices for intestinal organoid or tumoroid cultures include BME-1, BME-2 and collagen type I-A^8,24^. To investigate whether these alternative matrices could also support culture growth in low-viscosity (5%) matrix culture, matched organoids and tumoroids from a representative patient were grown for 14 days as LVM suspension cultures. As observed for Matrigel, BME-1, BME-2, and collagen type I-A all supported growth of both the organoids and tumoroids in LVM suspension culture (Supplementary Fig. 3).

In addition, LVM suspension conditions supported the three-dimensional growth of human cancer cell lines from the prostate (PC-3), breast (MDA-MB-231 and MCF-7), pancreas (BxPC-3), and lung (NCI-H520) cancer, suggesting that LVM suspension cultures will be useful for producing organoids from a variety of epithelial tissues (Supplementary Fig. 4).

### Organoid and tumoroid LVM suspension cultures recapitulate morphological development observed in dome cultures

Morphological development of organoids and tumoroids from single cells in the presence of solid support matrices has been extensively documented for normal colorectal epithelium and cancer, mirroring architectural features of the original tissue^2,8,10^. To determine whether comparable morphological development of organoids and tumoroids was maintained in LVM suspension culture, representative normal and cancer cultures were monitored for growth over a 14-day period in both the LVM suspension and dome culture conditions.

**Fig. 3:**
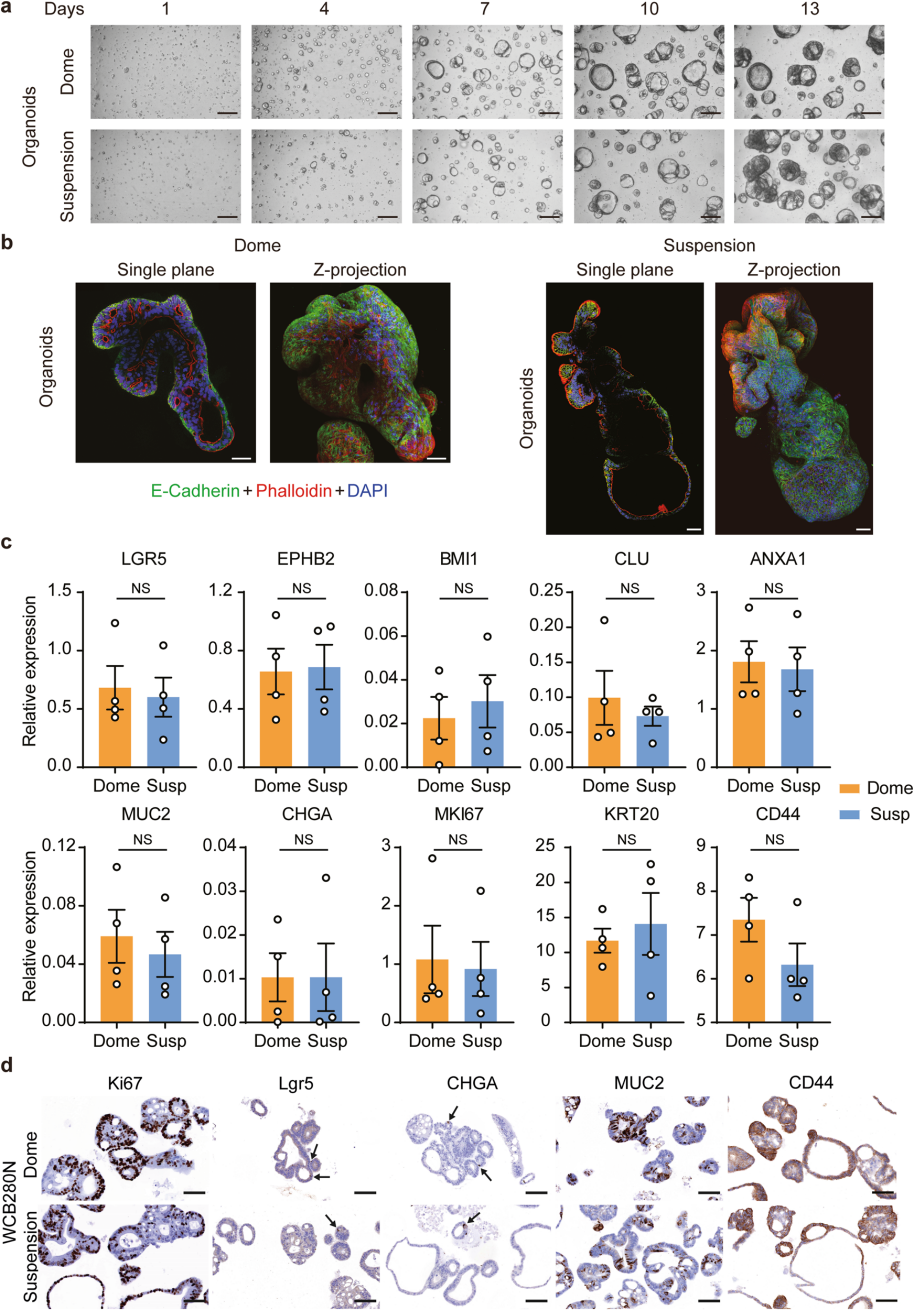
Colorectal organoids grown in low-viscosity matrix suspension recapitulate morphological development observed in dome culture. **a** Representative bright-field images of organoids grown in either LVM suspension or dome culture over a 14-day period; scale bars, 200 μm. **b** Immunofluorescence microscopy images of organoids grown as LVM suspension cultures for 21 days stained with phalloidin, DAPI, and E-cadherin antibody; scale bars, 50 μm. **c** qRT-PCR for organoids from four patients for markers of crypt base columnar stem cells (*LGR5, EPHB2*), quiescent stem cells (*BMI1*), revival stem cells (*CLU* and *ANXA1*), crypt base cells (CD44), transient amplifying cells (*MKI67*), goblet cells (*MUC2*), enteroendocrine cells (*CHGA*), and mature enterocytes and goblet cells (*KRT20*). **d** Immunohistochemistry for organoids from one representative patient stained for Lgr5, CD44, Ki67, MUC2, and CHGA; scale bars, 100 μm. Data (**c**) are plotted as mean ± s.e.m. Statistical significance was attributed to values of *p* < 0.05 as determined by the paired Student’s *t* test. NS, *p* > 0.05. N normal.

Normal colorectal organoids grown from single cells formed cystic-like structures after ~7 days in both the LVM suspension and dome culture conditions (Fig. 3a). The small normal organoids gradually ballooned out and after ~10–13 days began to undergo budding to form crypt-like extensions. Continuous expansion of the organoids in culture for more than two weeks resulted in the formation of a large mature organoid containing numerous crypt-like features (Fig. 3b, Supplementary Fig. 5). Normal organoid structures grown in LVM suspension conditions showed structural integrity with a high proportion of Ki67 positive cells within crypt buds (Supplementary Fig. 5), indicating sustained growth and regular morphogenesis during long-term expansion.

**Fig. 4:**
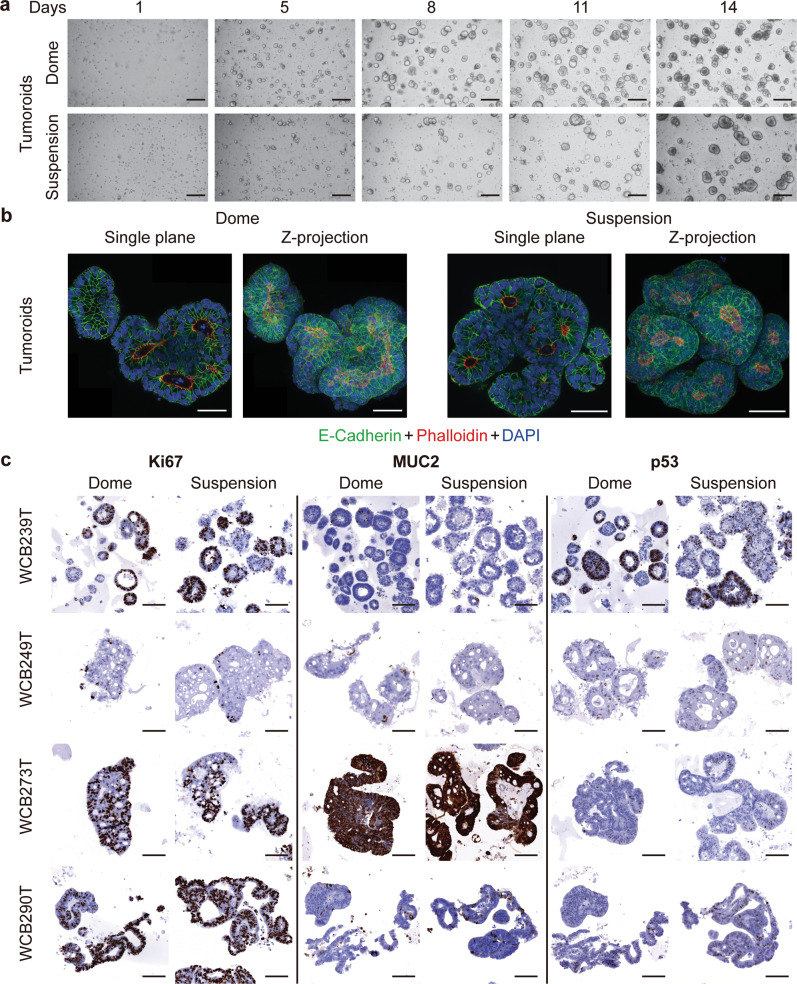
Colorectal tumoroids grown in low-viscosity matrix suspension recapitulate morphological development observed in dome culture. **a** Representative bright-field images of tumoroids grown in either LVM suspension or dome culture over a 14-day period; scale bars, 200 μm. **b** Immunofluorescence microscopy images of tumoroids grown as LVM suspension cultures for 21 days stained with phalloidin, DAPI and E-cadherin antibody; scale bars, 50 μm. **c** Immunohistochemistry for tumoroids from four patients stained for Ki67, MUC2, and p53; scale bars, 100 μm. T tumor.

For colorectal tumoroids grown in LVM suspension or dome culture, time varied from 7 to 20 days for single cells to form heterogeneous aggregates with admixed solid and cystic morphologies (Fig. 4a**)**. Over long-term culture in suspension, heterogeneous phenotypes and morphologies were maintained, with Ki67 positive cells interspersed throughout organoid structures (Supplementary Fig. 5).

Expression of E-cadherin was similar between organoids and tumoroids grown in LVM suspension and dome culture, with high expression at cell boundaries (Figs. 3b and 4b). For organoids, F-actin staining predominantly outlined the lumen when embedded in Matrigel matrix (basal-out polarity), whilst in LVM suspension culture some staining was observed in outwards facing regions (apical-out polarity) (Fig. 3b). Apical-out polarity was not observed in our limited number of tumoroids (Fig. 4b).

Major differentiated cell types of colonic crypts include intestinal stem cells, transient amplifying cells, enteroendocrine cells, goblet cells, and absorptive enterocytes. To assess whether organoids grown in LVM suspension culture mirrored dome cultures for expression of corresponding cell-type specific markers, matched organoid cultures from four patients were grown in both conditions for 14 days and examined by quantitative reverse transcription PCR (qRT-PCR) (Fig. 3c). qRT-PCR analysis of organoids revealed broad concordance of expression patterns between LVM suspension and dome cultures for markers of crypt base columnar stem cells (*LGR5, EPHB2*), quiescent stem cells (*BMI1*), revival stem cells (*CLU* and *ANXA1*), crypt base cells (CD44), transient amplifying cells (*MKI67*), goblet cells (*MUC2*), enteroendocrine cells (*CHGA*), and mature enterocytes and goblet cells (*KRT20*). Corresponding results were obtained for IHC analysis of matched LVM suspension and dome organoid cultures from three patients for Lgr5, CD44, Ki67, MUC2, and CHGA (Fig. 3d, Supplementary Fig. 6).

To evaluate concordance of marker expression between LVM suspension and dome cultures for tumoroids, IHC analysis was performed for Ki67, MUC2, and p53 for matched tumoroid cultures from eight patients. Matched tumoroids grown in both conditions exhibited similar patterns of marker expression for each patient with the anticipated heterogeneity in marker expression between patients (Fig. 4c, Supplementary Fig. 7).

Both colorectal organoids and tumoroids grown in LVM suspension culture maintained histopathological features similar to the original primary tissues (Supplementary Fig. 8–9).

### Colorectal tumoroids propagated in LVM suspension culture are representative of primary tumors at the genomic level

Twenty-six colorectal tumoroids were analyzed for mutations by whole-genome sequencing. In the absence of matched normal organoids, putative somatic mutations were identified for protein-coding exons by annotation against databases of known human germline variants, as well as five normal reference samples sequenced on the same platform.

**Fig. 5:**
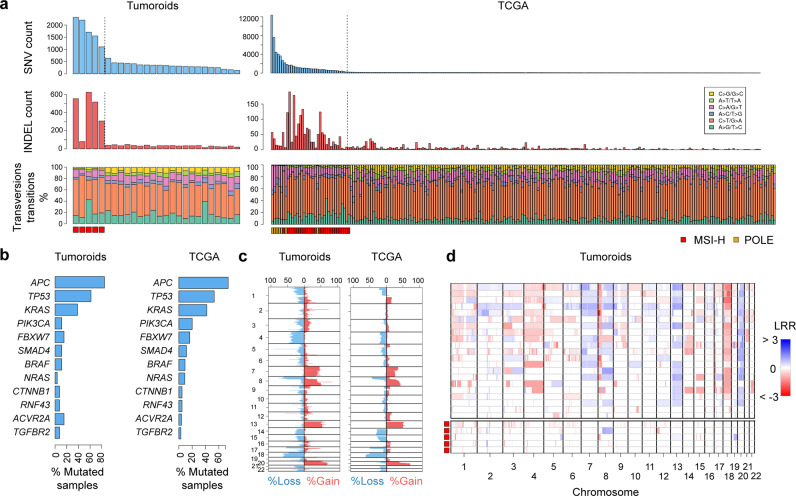
Global genomic alterations in human colorectal tumoroids. **a** Mutation profiles in 26 colorectal tumoroids and 224 TCGA colorectal cancers. Counts of SNVs and InDels, and proportions of nucleotide transitions and transversions are reported, split into distinct hypermutated and nonhypermutated cases. **b** Mutation frequencies of major colorectal cancer driver genes for tumoroids and TCGA-analyzed cancers. **c** Proportions of samples with relative DNA copy-number alterations for tumoroids and TCGA-analyzed cancers. **d** Genome-wide DNA copy-number aberrations for tumoroids stratified into nonhypermutated and hypermutated (MSI-H) cases.

Consistent with data on primary colorectal cancers reported by the TCGA^9^, the number of mutations varied widely among tumoroids, ranging from 1.7 to 30.1 per 10^6^ bases (Fig. 5a). Hypermutation with confirmed DNA mismatch-repair deficiency (dMMR) was evident for 19.2% (5/26) of tumoroids, similar to the 12.5% (28/224) of hypermutated cases among the TCGA cancers (*p* = 0.357). Tumoroids and TCGA cancers further showed similar mutation frequencies for major colorectal cancer-associated driver genes (Fig. 5b). Global DNA copy-number alterations in tumoroids mirrored those in TCGA cancers with frequent deletion of chromosome arms 8p, 17p (including *TP53*), and 18q (including *SMAD4*), and gain of chromosomes 7, 8q (including *MYC*), 13, and 20q (Fig. 5c). Consistent with the well-established associations in primary cancers, tumoroids with dMMR exhibited stable DNA copy-number profiles (Fig. 5d).

### LVM suspension culture facilitates colorectal organoid and tumoroid establishment

We next evaluated the utility of LVM suspension culture for organoid and tumoroid establishment as compared to donut cultures, where tissue fragments are seeded on top of a solidified ring of Matrigel matrix^23^, utilizing a consecutive series of 122 normal colorectal tissues and 91 cancer tissues.

**Fig. 6:**
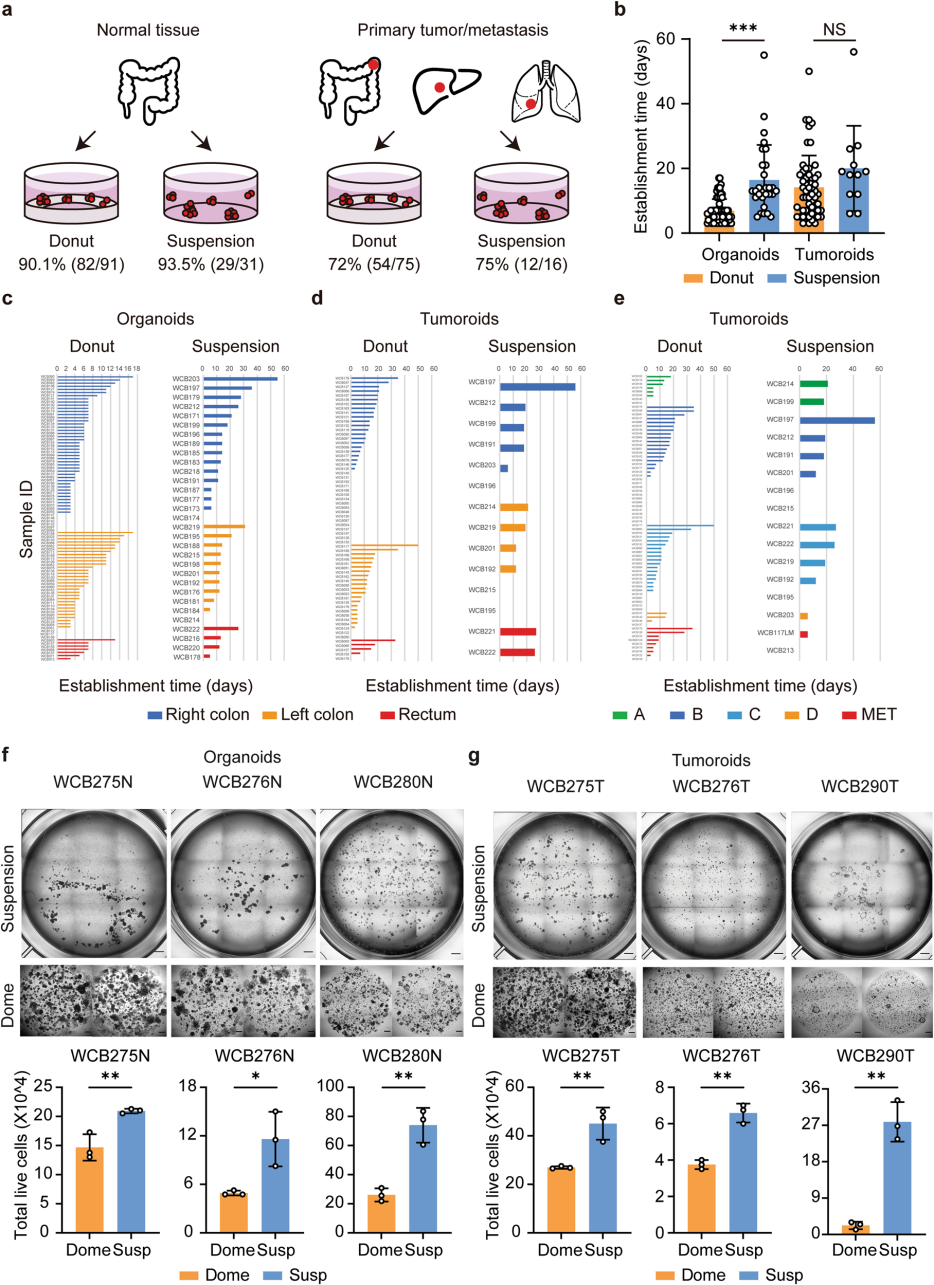
Establishment of colorectal organoids and tumoroids in low-viscosity matrix suspension culture. **a**–**b**, Comparison of (**a**) establishment rates and (**b**) times (passage 0 to 1) between LVM suspension and donut culture methods for organoids (*n* = 31 and *n* = 91, respectively) and tumoroids (*n* = 16 and *n* = 75, respectively). **c**–**e** Comparison of establishment times for organoids and tumoroids grown in LVM suspension or donut culture according to (**c**, **d**) location and (**e**) tumor stage. **f**–**g** Comparison of live-cell yield for (**f**) organoids and (**g**) tumoroids from three patients grown for 14 days in LVM suspension or dome culture; scale bars, 500 μm. Data (**b**, **f**, **g**) are plotted as mean ± s.d. Statistical significance (**b**, **f**, **g**) was attributed to values of *p* < 0.05 as determined by the Student’s *t* test. NS, *p* > 0.05, **p < 0.01, ***p < 0.001. N, normal; T, tumor.

For normal colorectal tissues establishment success was similar for both methods, with establishment rates of 93.5% (29/31) for LVM suspension and 90.1% (82/91; *p* = 0.728) for donut cultures (Fig. 6a). LVM suspension cultures sustained organoid growth for longer as compared to donut culture, allowing for an increased time to first passage with a mean of 16.4 days (s.d.=10.9) as compared to 6.9 days (s.d.=3.6; *p* < 0.001), respectively (Fig. 6b). Findings were similar for normal organoids derived from the right colon, left colon, and rectum (Fig. 6c, Supplementary Tables 3 and 4).

For tumoroids, establishment success was also similar for the LVM suspension and donut culture methods, with establishment rates of 75.0% (54/75) and 72.0% (12/16, *p* = 1.000), respectively (Fig. 6a). As observed for normal tissues, time to first passage for tumoroids could be prolonged in LVM suspension culture (mean = 20.0 days; s.d.=13.2) as compared to donut culture (mean = 14.1 days; s.d.=9.9 days), although this difference was not statistically significant (*p* = 0.084) (Fig. 6b). Similar patterns were observed irrespective of location or tumor stage (Fig. 6d, e, Supplementary Tables 3 and 4).

To compare live-cell yields for organoids or tumoroids established in LVM suspension or dome culture, normal crypt, or cancer tissue fragments (6000 fragments, 1.5 ml of medium/well) from three colorectal cancer patients were suspended in media containing 5% Matrigel or embedded in solid Matrigel. Cultures were grown over a 14-day period with media changes every 2 days. Both the organoids and tumoroids grown in LVM suspension culture produced significantly higher yields of viable cells compared to the dome cultures (organoids: 1.4–2.8-fold increase; tumoroids: 1.7–11-fold increase) (Fig. 6f, g).

Notably, LVM suspension conditions also supported the establishment of mouse colon and small intestinal organoid cultures, indicating cross-species versatility of this culture approach (Supplementary Fig. 10).

### Scalable expansion of intestinal organoids and tumoroids in bioreactor tubes

Current organoid and tumoroid culture techniques, that require a solid support matrix, limit the automation and scalability of organoid production. To examine whether the LVM suspension conditions were amenable to bioreactor applications, independent patient-derived organoids (*n* = 2) and tumoroids (*n* = 3), were suspended as single cells (200,000 cells) in 50 ml Bioreactor Tubes containing 7 ml of medium with 5% Matrigel. Organoids and tumoroids were cultured in duplicate with media changes (50:50) and gentle agitation by pipetting every 2–5 days.

**Fig. 7:**
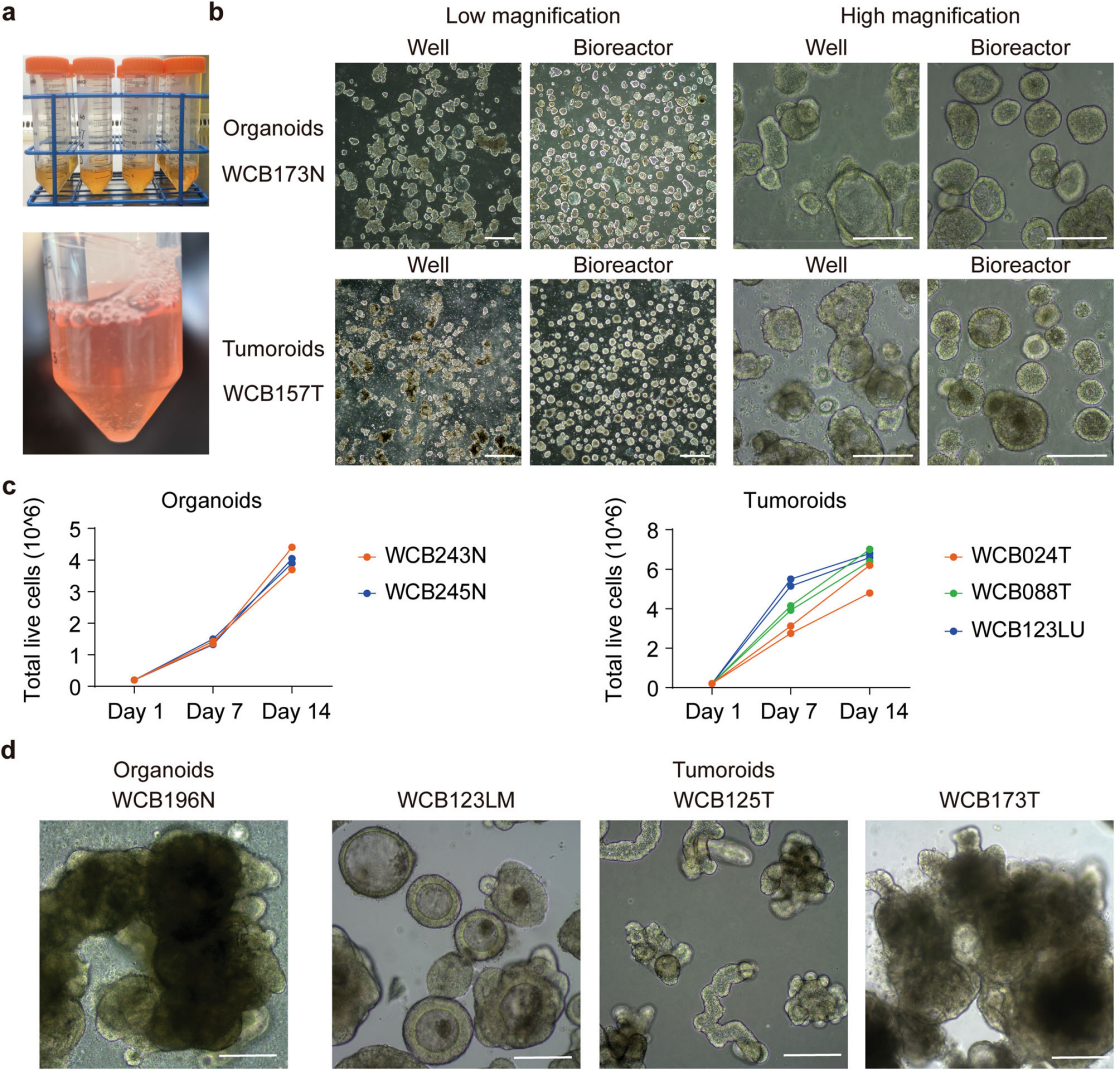
Scalable expansion of colorectal organoids and tumoroids in bioreactor tubes. **a** Image of Corning™ Mini Bioreactor Centrifuge Tubes used for culture expansion. **b** Representative images of organoids and tumoroids grown as LVM suspension cultures in 6-well plates or bioreactor tubes over a 14-day period; low-magnification images, scale bars, 500 μm; high-magnification images, scale bars, 200 μm. **c** Quantification of normal and cancer organoid growth in bioreactor tubes. A total of 200,000 cells were seeded in 7 ml LVM suspension culture medium per tube; two independent normal and three independent cancer organoid cultures were assayed in duplicate. **d** Representative images of normal and cancer organoids grown in bioreactor tubes for 4–8 weeks, resulting in substantial enlargement of organoids and/or organoid aggregates; scale bars, 200 μm. N, normal; T, tumor; LM, liver metastasis; LU, lung metastasis.

Bioreactor cultures of organoids and tumoroids maintained morphological features similar to LVM suspension cultures in 6-well plates over a 14-day period (Fig. 7a, b), and demonstrated substantial increase in yield of viable cells achieving 3.70 × 10^6^–4.05 × 10^6^ cells for normal organoids and 4.80 × 10^6^–7.00 × 10^6^ cells for tumoroids (Fig. 7c). Continuous expansion of organoids and tumoroids in bioreactor tubes for 4–8 weeks resulted in progressive enlargement and/or aggregation of organoids and tumoroids. For normal organoids, long-term bioreactor culture generated organoids with hundreds of crypt-like protrusions, while for tumoroids complex heterogeneous morphologies were obtained (Fig. 7d). This bioreactor method; therefore, enables substantial organoid expansion within a low passage number, which is necessary for maintenance of organoid and tumoroid genomic fidelity and scalable production.

### LVM suspension culture of colorectal tumoroids in 384-well format

To evaluate the utility of LVM suspension cultures for assay miniaturization, we determined whether patient-derived colorectal cancer cells could be seeded and robustly cultured as tumoroids in a 384-well format. To facilitate automated dispensing of the LVM cell suspension, the Matrigel concentration was reduced to 3%; a seeding density of 3000 cells/well (in 60 μl media) was found to produce ~150–300 distinct tumoroids suitable for imaging.

**Fig. 8:**
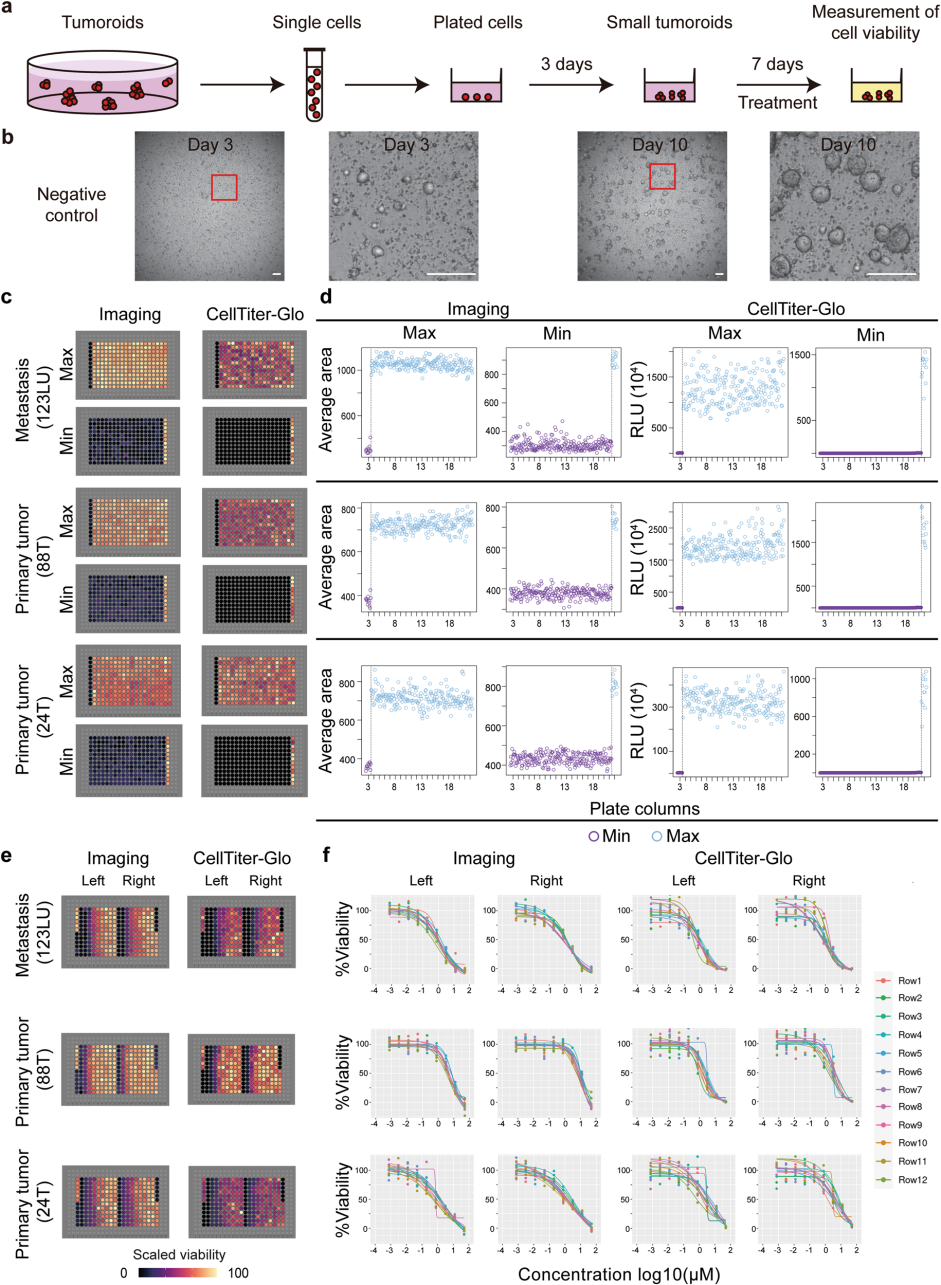
Plate uniformity of tumoroid viability assays with low-viscosity matrix suspension culture in 384-well format. **a** Schematic of the workflow for tumoroid viability assessment. Established tumoroids were dissociated and seeded as single cells, grown into small tumoroids over 3 days and treated with drug for 7 days with daily bright-field *z*-stack imaging. Cell viability was determined by both image analysis and CellTitre-Glo 3D assays. **b** Representative images of vehicle-treated tumoroids in 384-well format on day 3 and day 10 of the assay, scale bars, 100 μm. **c**–**d** Tumoroids from three patients were examined for uniformity of maximum (Max) and minimum (Min) signals; (**c**) plate signals were visualized by heat maps, and (**d**) raw signals of mean tumoroid size or relative luminescence units (RLU) were plotted against the respective plate column. **e**-**f** Tumoroids from three patients were examined for uniformity of drug dose-response curves; (**e**) plate signals were visualized by heat maps, and (**f**) four-parameter logistic regression was used to fit drug dose-response curves for each side of each plate (left and right); each color represents a different plate row. T tumor, LU lung metastasis.

To examine tumoroid growth in 384-well format, single-cell suspensions from three representative tumors were dispensed using a Mantis Liquid Handler. Plates were imaged every 24 h for 10 days on an automated Nikon Eclipse Ti2 Inverted Microscope System with an integrated tissue culture incubator to measure organoid size followed by determination of cell viability (ATP consumption) using CellTitre-Glo 3D reagent (Fig. 8a and Supplementary Fig. 11a). In concordance with larger plate formats, tumoroids formed within 3 days and expanded over the subsequent 7-day period without requiring media changes (Fig. 8b). Due to media evaporation over the 10-day assay period, an edge effect was observed with reduced tumoroid growth in the outer two rows (A & B and O & P) and columns (1 & 2 and 23 & 24) (Supplementary Fig. 11b); in subsequent assays these outer wells were omitted from analysis.

Plate uniformity in the 384-well format was evaluated for maximum (Max) signals, minimum (Min) signals, and drug dose-dependent midpoint (Mid) signals (Fig. 8c–f). To determine Max signals, tumoroids were established for 3 days, treated with 0.5% DMSO (vehicle control) using a JANUS Automated Liquid Handling Workstation with a 384 pin tool, and incubated for 7 days. Informed by our previous studies on colorectal cancer cell lines^25^, Min signals were determined using the proteasome inhibitor bortezomib as a cell-killing control with complete growth inhibition observed at 1 μM (Supplementary Fig. 12). To evaluate dose-dependent Mid signals, regorafenib was titrated across each half of the assay plate in a nine-step, four-fold dilution series starting from 50 μM, generating a total of 24 drug dose-response curves. Tumoroid viability was determined by both the image analysis and CellTiter-Glo 3D luminescence measurements. Mean tumoroid size and luminescence signals were visualized as heatmaps for each plate and plotted by wells for each plate row from left to right or as drug dose-response curves (Fig. 8).

Performance metrics for plate uniformity were compared between the image analysis and CellTitre-Glo 3D assays. Image analysis produced CVs of 1.9–5.9% for Max signals, 2.2–15.4% for Min signals, and 4.4–9.8% for Mid signals, well below the required compliance threshold of 20% for high-throughput cell-based screens (Supplementary Table 5)^26^. Similarly, all signal windows were greater than 2 (range 4.3–29.0), and all robust Z’ factors were greater than 0.50 (range 0.50 to 0.82), indicating an excellent quality of the tumoroid assays for imaging. We observed an overall poorer performance for CellTiter-Glo assays (Supplementary Table 6). CVs ranged from 8.2 to 24.0% for Max signals, 13.8 to 89.2% for Min signals, and 18.0 to 29.3% for Mid signals. Some signal windows were less than 2 (range 1.13–9.02), and some robust Z’ factors were less than 0.50 (range 0.16–0.77).

For both the image analysis and CellTitre-Glo 3D assays, the ED50 mean fold-changes between plates were all acceptable at less than two-fold (imaging: range 1.26–1.67; CellTitre-Glo 3D: range 1.09–1.73; Supplementary Tables 5 and 6)^27^.

ED_50_ values were marginally different (>2 fold) between the image analysis and CellTitre-Glo 3D assays for two out of three tumoroids with higher potency estimates for viability measurements based on ATP consumption (Supplementary Tables 5 and 6). Differential drug potency estimates for imaging and nonspecific metabolic activity assays are well-documented^28,29^, and regorafenib has been shown to impair mitochondrial function and decrease cellular ATP levels^30^.

### Utility of LVM suspension culture for tumoroid diagnostic testing and high-throughput drug screening

To demonstrate the utility of LVM suspension culture for diagnostic testing and high-throughput screening applications, we evaluated the reproducibility of imaging-based drug sensitivity testing of tumoroids for clinically relevant agents including 5-FU (pyrimidine analogue), oxaliplatin (DNA intercalating agent), SN-38 (active metabolite of the topoisomerase I inhibitor irinotecan), regorafenib (multikinase inhibitor), and TAS-102 (thymidine-based nucleic acid analogue and a thymidine phosphorylase inhibitor). Tumoroids were grown over the course of 3 days, followed by automated addition of drug dilutions from preprepared master compound plates. Drug dilutions covered the physiological concentrations of 5-FU (*C*_max_ ≈ 7.5 µM)^31^, oxaliplatin (*C*_max_ ≈ 5.0 µM)^32^, SN-38 (*C*_max_ ≈ 0.14 µM)^32^, regorafenib (*C*_max_ ≈ 7.3 µM) (NCT01853046), and TAS-102 (*C*_max_ ≈ 18.2 µM) (AusPAR 2018) observed in patients. All drugs were assayed in duplicate in a nine-step, four-fold dilution series with daily imaging for 7 days. 0.5% DMSO and bortezomib (1 µM) served as negative (vehicle) and positive (PDTO killing) controls, respectively. Drug responses were evaluated using growth rate-adjusted (GR) measurements based on comparing growth rates in the presence and absence of drug. Parametrization of GR data yields GR_50_, GR_max_, and GR_aoc_ values that are largely independent of division rate and assay duration^33^.

**Fig. 9:**
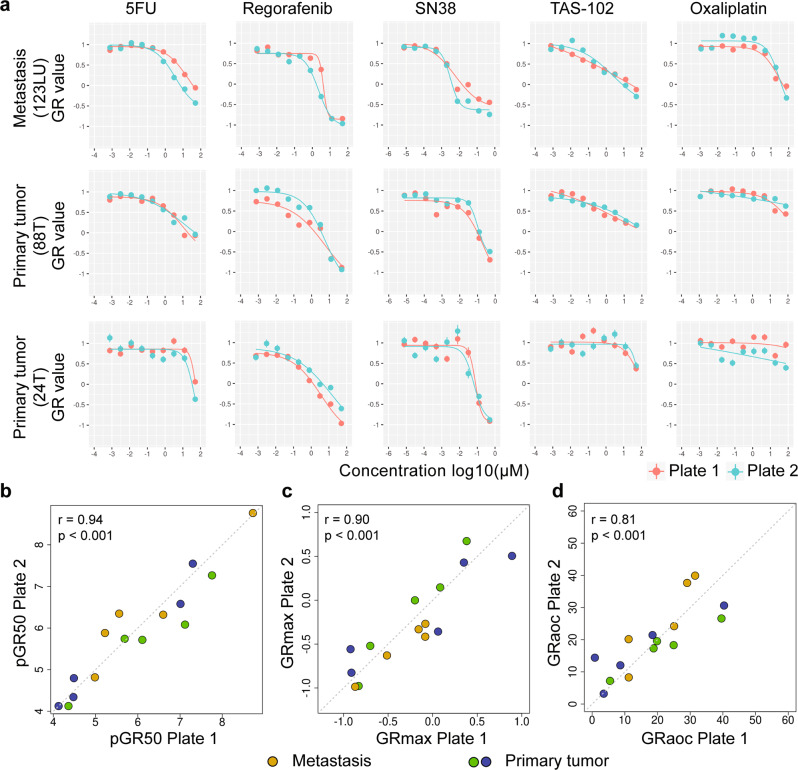
Reproducibility of imaging-based drug sensitivity testing of tumoroids for clinically relevant agents including 5-fluorouracil (5-FU), oxaliplatin, SN-38, regorafenib and TAS-102. **a**, Tumoroids from three patients were assayed in two independent runs consisting of duplicate drug titrations (corresponding 200–400 organoids) for 5-FU, oxaliplatin, SN-38, regorafenib and TAS-102. Tumoroid sizes were calculated across duplicate wells and plotted as mean ± s.e.m. Four-parameter logistic regression was used to fit drug dose-response curves. **b**–**d** Between run correlations for (**b**) pGR50 (**c**) GR_max_ and (**d**) GR_aoc_ estimates. Statistical significance (**b**–**d**) was attributed to values of p < 0.05 as determined by the t test and Pearson’s correlation coefficient (r). T, tumor; LU, lung metastasis.

Two independent runs were performed for three colorectal tumoroids. Four-parameter logistic regression was used to fit drug dose-response curves, with high concordance of curve fits evident between runs for all five drugs (Fig. 9a). Accordingly, replicate experiments showed strong correlations for pGR_50_, GR_max_ and GR_aoc_ estimates, with Pearson’s correlation coefficients of 0.94, 0.90, and 0.81, respectively (*p* < 0.001 for all comparisons) (Fig. 9b–d).

## Discussion

Although patient-derived colorectal epithelium and cancer cells can be cultured in vitro as organoids and tumoroids in solid support matrices such as Matrigel^2,10,11^, the use of solid matrices poses technical challenges for the automation and scalability of cultures necessary for downstream experimental and preclinical applications^8,13,15,20–22^. In addition, culture growth in a solidified matrix is limited due to solid stress accumulation, oxygen, and nutrient delivery^19^, necessitating frequent passaging to maintain and expand cultures. Our results show that solid support matrices can be replaced with a LVM suspension preparation to enable efficient establishment, propagation, and expansion of organoids and tumoroids from the human large intestine. We further show that tumoroid LVM suspension cultures are amenable to assay miniaturization and imaging-based diagnostic tests and automated high-throughput screening applications. LVM suspension culture thus constitutes a parallel method to classic submerged solid matrix culture and air-liquid interface culture where organoids containing both the epithelial cells and surrounding stroma are grown as a cohesive unit directly from tissue fragments^34^.

Cell embedment in solid matrices is the current mainstay for the growth and propagation of patient-derived intestinal organoids and tumoroids^2,10,11^. We have demonstrated that solid support matrices can be replaced with a LVM (3–5% Matrigel) suspension preparation to enable the robust culture of both the colorectal organoids and tumoroids. Organoids achieved establishment rates of 93.5% and propagation rates of 87.0% in IntestiCult medium tailored to the niche requirements for growth of normal intestinal epithelium^5,6^, while tumoroids achieved establishment rates of 75.0% and propagation rates of 75.9% in reduced medium lacking Wnt agonists, BMP, TGF-β, and p38 inhibitors. The lower culture efficiency for tumoroids grown in reduced medium as compared to organoids grown in IntestiCult medium is consistent with previous studies of colorectal tumoroids (*n* > 30) employing similar reduced media conditions, with reported establishment rates ranging from 58 to 86%^12,14,15,35,36^. Combinatorial culture conditions with varying addition of Wnt agonists (Wnt3A/R-spondin1), oxygen concentration, and a p38 inhibitor have been shown to increase tumoroid culture efficiencies to near 100%^10^, suggesting a margin for further optimization of tumoroid LVM suspension culture. Similarly the use of IGF-1 and FGF-2 in the culture medium of human intestinal organoids has been shown to promote secretory cell differentiation without impairing organoid growth^37^. Further improvements to media conditions may be achieved by the replacement of classic niche factors with emerging biomimetic compounds^38,39^.

LVM suspension cultures could be easily harvested, with floating live organoids readily separated from tissue or cell debris precipitating at well bottoms, and could be dispensed using liquid-handling systems without additional cooling requirements to keep Matrigel in a liquid state. Organoid and tumoroid growth in LVM suspension were also supported in other biological scaffolds including BME-1, BME-2, and collagen type 1-A, although we did not explore the use of synthetic hydrogels which will ultimately be required for producing clinical-grade organoid cultures that comply with good manufacturing practice^4^. Importantly, LVM suspension culture enabled scalable expansion of organoids in Bioreactor tubes, achieving a 20- to 35-fold increase of live-cell numbers within 14 days, required to produce sufficient cells for high-throughput assay applications. Higher yields may be achieved in larger-scale bioreactor formats, such as continuous stirred tank bioreactors, although this was not evaluated in this study.

Organoids and tumoroids grown in LVM suspension recapitulated the morphological development observed in solid matrix cultures, including the growth trajectories and marker expression, and retained features of the original normal or cancer tissues. Nonetheless, one point of difference for normal organoids was that whilst these tended to show a basal-out polarity when embedded in Matrigel, these exhibited variable basal-out and apical-out polarity in LVM suspension culture. This observation is consistent with a previous study showing the transition of intestinal organoids from basal-out to apical-out polarity upon removal of ECM scaffold proteins^40^.

The LVM derived tumoroids further reflected the genetic heterogeneity of primary colorectal cancers, with similar mutation and DNA copy-number profiles as compared to TCGA-analyzed colorectal cancers. Corresponding findings have been reported for colorectal tumoroids established in solid Matrigel^8,10–14^, although tumoroids with chromosome instability^41^ or DNA mismatch-repair deficiency^42^ continuously accrue chromosome mis-segregation errors or mutations during culture. Organoids and tumoroids propagated in LVM suspension culture could be biobanked and recovered. LVM suspension culture is therefore suited for the establishment of biorepositories of CRC models representing the spectrum of clinical disease, enabling mechanistic and translational studies.

We have demonstrated the applicability of our LVM suspension culture method for tumoroid high-throughput assay applications, establishing a semiautomated drug testing platform in 384-well format with robotic solutions for cell seeding, compound administration, and viability read-outs. Interexperimental variability was reduced by optimizing critical experimental parameters, including controls, cell seeding density, and edge effects. In miniaturized 384-well format, tumoroids readily formed from single cells and expanded over a 10-day period, with drug addition on day 3 allowing to monitor drug responses over a 7-day period, capturing multiple cell divisions. Time-resolved 3D image analysis facilitated implementation of growth rate-adjusted drug response metrics^33^. The image analysis was more robust and reliable than metabolic activity assays, passing relevant criteria of assay performance. Compared to previous high-throughput platforms for tumoroid drug screening using solid Matrigel^8,13,15,20–22^, our LVM suspension culture-based platform offers advantages in terms of ease of assay set up and reduction in cost.

Several studies have reported that colorectal tumoroids can forecast patient responses to neoadjuvant chemoradiation and anticancer drugs, although data are still emerging and utility may be limited to certain clinical scenarios^14,15,36^. To enable the use of colorectal tumoroids for the guidance of clinical decisions and personalized medicine, the time needed for preparing a tumoroid culture for drug screening is a key factor. In LVM suspension culture, starting with a 5 mm^3^ tissue specimens, mean establishment and propagation times for tumoroids were 20.0 and 21.5 days, respectively, allowing for an assay reporting time of 6–8 weeks. Improvements to this turn-around time may be achieved with further assay miniaturization, such as the transition from 384- to 1536-well formats to reduce the required cell input number; this will be critical for the development of diagnostic applications using tumor samples from core needle biopsies.

Organoid and tumoroid cultures are being established from an ever-increasing number of human tissues, as well as laboratory and domesticated animals^43,44^. While this study focused on human cultures from the large intestine, our LVM suspension culture approach was also applicable to the establishment of murine colon and small intestinal organoids, underscoring the cross-species versatility of this method. Moreover, our LVM suspension culture approach supported the three-dimensional growth of human cancer cell lines of the prostate, breast, pancreas, and lung, suggesting that this method is suitable for the analysis of the cell biology and drug sensitivity of other epithelial tissues. LVM suspension cultures simplified tumoroid experimental manipulation, molecular annotation, and automated image analysis to assess drug sensitivity. Other applications which may be facilitated by this approach include genetic manipulation, co-culture with immune cell populations, which is pertinent to the development of cancer immunotherapies, propagation and study of intestinal viruses, and other pathogens, as well as production of biological products from intestinal tissue and regenerative medicine applications.

In summary, this study describes a reliable LVM suspension culture method for the growth of organoids and tumoroids from the human large intestine. The LVM suspension culture method provides a tool for the application of patient-derived intestinal tissue and may be applicable to organoid and tumoroid production from diverse epithelial tissues.

## Methods

### Patient specimens

Tumor and adjacent normal tissue samples were obtained from patients with colorectal adenocarcinoma recruited at the Western Health Hospital Footscray, Eastern Health Hospital Box Hill, Northern Health Hospital Epping, and Royal Melbourne Hospital Parkville in Australia between 2017 and 2020. This study was conducted in accordance with the Declaration of Helsinki, the NHMRC Statement on Ethical Conduct in Human Research, and Institutional Human Research Ethics approval (HREC 2016.249, Walter and Eliza Hall Institute of Medical Research). All patients gave informed consent. Tumoroids and organoids were generated from respective tumor tissue and adjacent normal tissue from the resection margin from patients undergoing resection of primary tumors. For patients undergoing resection of metastatic disease, tumoroids were generated from tissue taken from the site of the metastasis. Specimens with a volume of greater than 5 mm^3^ were collected at surgery and placed into a collection medium containing DMEM/F12 (Life Technologies, 11320082), 250 U/ml penicillin-streptomycin (Life Technologies, 15140122), 50 µg/ml gentamycin (Merck, G1397), 100 µg/ml kanamycin (Merck, K0254), 2.5 µg/ml amphotericin B (Merck, A2942) and 100 U/ml nystatin (Merck, N1638), and stored for up to 72 h at 4 °C before processing.

### Cancer cell lines

The following cancer cell lines were used: Human prostate cancer cell line PC-3 (ATCC, CRL-1435), breast cancer cell lines MCF7 (ATCC, HTB-22) and MDA-MB-231 (ATCC, HTB-26), pancreatic cancer cell line BxPC3 (ATCC, CRL-1687) and lung cancer cell line NCI-H520 (ATCC® HTB-182. All cell lines were authenticated by STR analysis using the GenePrint 10 System (Promega) at the Australian Genome Research Facility (AGRF).

### Mouse specimens

Colon and small intestinal tissues were harvested from a 8.5 week old C57/B16 male mouse for organoid generation (AEC 2018.038, Walter and Eliza Hall Institute of Medical Research).

### Organoid and tumoroid generation

Human and mouse tissues were treated with 0.10% sodium hypochlorite in phosphate-buffered saline (PBS) for 10–20 min at room temperature and washed with ice-cold PBS. For normal intestinal tissues, specimens were incubated in 3 mM EDTA chelation buffer (Sigma-Aldrich, E5134) containing 100 µM dithiothreitol (Merck, 10197777001) for 30–60 min at room temperature, transferred into PBS and shaken vigorously to release crypts. Crypts were collected by centrifugation, digested with 0.1 mg/ml dispase (Life Technologies, 17105-041) in DMEM/F12 to produce fragments of crypts, and the digested samples were washed with DMEM/F12. Organoids from crypt fragments were grown using the three culture methods described below in IntestiCult Organoid Growth Medium (Human or Mouse) (Stemcell Technologies, 06010 or 06005) containing 10 μM Rho-kinase inhibitor Y27632 (Stemcell Technologies, 72308) 100 U/ml penicillin-streptomycin (Life Technologies, 15140122) and 100 µg/ml primocin (Invivogen, ant-pm-1).

Tumor samples were digested with 0.1 mg/ml dispase and 200 U/ml collagenase IV (Life Technologies, 17104019) in DMEM/F12 using a gentleMACS™ Dissociator (Miltenyi Biotec, 130-093-235), washed with DMEM/F12 and fragments were collected by centrifugation. The tissue fragments were filtered through a 70 μm cell strainer (Bio-Strategy, BDAA352350), washed with DMEM/F12 and tumoroids grown using the three culture methods described below in reduced medium containing DMEM/F12, HEPES, 1x B-27 supplement, 1x N2 supplement, 10 mM nicotinamide, 1 mM N-acetyl-L-cysteine, 20 ng/ml recombinant human basic FGF (Life Technologies, PHG0263), 50 ng/ml recombinant human EGF, 100 U/ml penicillin-streptomycin and 100 µg/ml primocin; 10 µM Y27632 was added at initial seeding.

### Organoid and tumoroid culture methods

Organoids and tumoroids were grown in a fully humidified CO_2_ incubator (Thermo Fisher Scientific) at 37 °C with 5% CO_2_. Culture media were as described above with replacement of primocin by 100 µg/ml normocin. Three culture methods were used in this study:

### Donut culture^24^

For establishment, 15 μl of Matrigel matrix (Bio-strategy, BDAA354234), chilled at 4 °C, was used to generate a ring around the circumferences of a well in a 96-well plate. The Matrigel ring was solidified at 37 °C for 30 min and then overlaid with a culture medium containing normal crypt or tumor tissue fragments (50–100 µm) with a density of 300 fragments in 150 µl/well. The culture medium was changed every 2–3 days.

### Dome culture

For establishment, normal crypt or tumor tissue fragments were suspended in Matrigel matrix and plated as 30 µl domes (each dome containing 3000 fragments) in 6-well plates with a density of 2–8 domes per well. Matrigel domes were solidified by incubation at 37 °C for 30 min, followed by the addition of respective organoid or tumoroid culture media (3–3.5 ml/well). The culture medium was changed every 2–3 days. For passaging, organoids or tumoroids (diameter of 100–300 μm) were harvested by scraping domes off wells followed by digestion with TrypLE Express enzyme (Thermo Fisher Scientific, 12604021) for 10–20 min at 37 °C. Digestion was terminated with 1% BSA in DMEM/F12 and organoids or tumoroids were pelleted by centrifugation. Pellets were resuspended in culture medium and mechanically broken into fragments by pipetting with a T200 pipette tip or to single cells by pipetting with a 26 G needle. Organoid or tumoroid fragments (50–100 µm) or single cells were suspended in Matrigel matrix and plated as 30 µl domes (each dome containing 100 fragments or 100,000 cells) in 6-well plates with a density of eight domes per well. Matrigel domes were solidified by incubation at 37 °C for 30 min, followed by addition of respective organoid or tumoroid culture media (3.5 ml/well). The culture medium was changed every 2–3 days.

### LVM suspension

For the establishment, normal crypt or tumor tissue fragments (50–100 µm) were suspended in media containing 5% Matrigel and plated in 6-well or 24-well suspension plates (Interpath, 657185, 662102) with a density of 24,000 or 6000 fragments in 3.5 or 1 ml/well, respectively. For passaging, organoids or tumoroids (diameter of 100–300 μm) were aspirated with a pipette, digested with TrypLE Express enzyme, and mechanically broken into fragments or single cells as described above. Organoid or tumoroid fragments (50–100 µm) were suspended in media containing 3–10% Matrigel or other 5% matrix preparations (BME-1 (Basement Membrane Extract, Type 1, PathClear, Cultrex, 3432-010-01), BME-2 (Basement Membrane Extract, Type 2, PathClear, Cultrex, 3532-010-02) or collagen type I-A (Cellmatrix Collagen Type I-A, Nitta Gelatin Inc., 631-00651), and plated in 6-well suspension plates (Interpath, 657185) or Corning™ Mini Bioreactor Centrifuge Tubes (Corning, 431720). Plating densities were 1000 organoid or tumoroid fragments or 100,000 cells in 3.5 ml/well in 6-well plates, 25,000 cells in 1 ml/well in 24-well plates, and 200,000 or 500,000 cells in 7 or 17.5 ml/tube in bioreactor tubes. The culture medium was changed every 2–3 days with agitation of organoids by pipetting to prevent organoids from adhering to the bottom surface of wells or the tube bioreactors.

### Organoid mycoplasma testing and biobanking

Organoid and tumoroid cultures were tested for the absence of mycoplasma using the LookOut Mycoplasma PCR Detection Kit (Sigma-Aldrich, MP0035-1KT). For biobanking, small (50 to 100 μm) organoids or tumoroids were harvested, washed and suspended with DMEM/F12, combined with CryoStor 10 medium (STEMCELL Technologies, 7930), frozen in a Corning CoolCell LX Cell Freezing Container (Biocision, BCS-405) at -80 °C and transferred into liquid nitrogen for long-term storage.

### Bright-field microscopy

Bright-field microscopy to document organoid and tumoroid growth over time was performed on a Nikon Eclipse TS100 microscopy system with 2× or 10× objectives, a TrueChrome camera (Tucsen) and TCapture software (version 5.1.1.0) or, for z-stack projection images, on a Nikon Eclipse Ti-U microscopy system with 4× or 10× objectives, a DS-Ri2 camera (Nikon) and NIS-Elements BR software (version 4.40.00, 64-bit).

### Histology and immunohistochemistry

Organoids and tumoroids were harvested, mixed with HistoGel warmed at 55 °C (Themo Fisher Scientific, 22-110-678), pipetted onto 6-well plates to generate domes and solidified at 4 °C for 15 min. The domes were fixed in 10% formalin for 30 min and covered in PBS at 4 °C before embedding in paraffin. Normal and tumor tissue samples from patients with colorectal cancer were embedded in Tissue-Tek O.C.T Compound (Emgrid, 4583) and frozen rapidly using an isopropyl bath surrounded by dry ice. Specimen sections were prepared for hematoxylin and eosin (H&E) staining.

For immunohistochemistry, sections were prepared from formalin-fixed, paraffin-embedded specimens and stained with antibodies against Ki67 (clone MIB-1, DAKO, M7240, 1:100), Lgr5 (clone OTI2A2, Thermo Fisher Scientific, TA503316, 1:300), Chr-A (CHGA) (clone C-12, Santa Cruz Biotechnology, sc-393941, 1:50), Mucin 2 (clone CCP58, Santa Cruz Biotechnology, sc-7314, 1:100), HCAM (CD44) (Santa Cruz Biotechnology, sc7297, 1:50) and p53 (clone DO-1, Santa Cruz Biotechnology, sc-126, 1:200) on a Dako Omnis platform. Sections were treated with Retrieval Solution, Low pH (DAKO, K800521-2) or High pH (DAKO, K800421-2) at 97 °C for 30 min. Sections were incubated with primary antibody for 1 h and secondary antibody for 30 min followed by substrate chromogen (DAB) (DAKO, GV82511-2) treatment for 10 min and counterstaining with haematoxylin. The stained slides were scanned on a 3D Histech Panoramic Scan II histology scanner (3DHISTECH Ltd.) and examined using CaseViewer Software (3DHISTECH Ltd.).

### RNA extraction and quantitative reverse transcription PCR

Total RNA was extracted from organoids with the ISOLATE II RNA Mini Kit (Bioline, BIO-52073) or the RNeasy Micro Kit (QIAGEN, 74004). Total RNA was reverse transcribed and amplified using the TransPlex® Whole Transcriptome Amplification Kit (Sigma-Aldrich, WTA1). PCR product was purified using GenElute™ PCR Clean-Up Kit (Sigma-Aldrich, NA1020). Samples for qRT-PCR were prepared with the SensiFAST™ SYBR® No-ROX Kit (Bioline, BIO-98020). qRT-PCR was performed on the Applied Biosystems™ 7500 Fast Dx Real-Time PCR Instrument and relative mRNA expression of genes was normalized to a housekeeping gene, PBGD, using 2^−ΔCt^ method (2^−ΔCt^ =$\,{2}^{-({{{{{{\rm{ct}}}}}}}_{{{{{{\rm{gene}}}}}}}-{{{{{{\rm{ct}}}}}}}_{{{{{{\rm{PBGD}}}}}}})}$). The following primers were used: LGR5, 5’-CAGCGTCTTCACCTCCTACC-3’ and 5’-TTTCCCGCAAGACGTAACTC-3’; EPHB2, 5’-CTTCGAGGCCGTTGAGAAT-3’ and 5’-ATTGCGGCAGACACAGTTG-3’; BMI1, 5’-CTCGCATTCATTTTCTGCTG-3’ and 5’-ACACACATCAGGTGGGGATT-3’;CLU, 5’-CGGATGAAGGACCAGTGTG-3’ and 5’-TTCCTGGTCAACCTCTCAGC-3’; ANXA1, 5’-GGCCTTGGAACTGATGAAGA-3’ and 5’-CAAAGCGTTCCGAAAATCTC-3’; CD44, 5’-CCCAGATGGAGAAAGCTCTG-3’ and 5’- GTTGTTTGCTGCACAGATGG-3’, MKI67, 5’-CAGTTCCACAAATCCAACACA-3’ and 5’-GCTGGCTCCTGTTCACGTAT-3’; MUC2, 5’-CTGCTGACCATCAAGGATGA-3’ and 5’-AGGCATCGCTCTTCTCAATG-3’; CHGA, 5’-CGGATCCTTTCCATTCTGAG-3’ and 5’-TCATCTTCAAAACCGCTGTG-3’; KRT20, 5’-GGAGCAGTCCAACTCCAAAC-3’ and 5’-GAGCATTTTGCAGTTGAGCA-3’; and PBGD, 5’- CACCACAGGGGACAAGATTC-3’ and 5’-ATGGTGAAGCCAGGAGGAA-3’.

### Immunofluorescence microscopy

Organoids and tumoroids were harvested, washed with PBS, fixed with 10% formalin for 60 min at 4 °C, permeabilized in 0.5% Triton X-100 in PBS for 30 min and blocked in 1% BSA in PBS overnight at 4 °C. Organoids and tumoroids were thoroughly rinsed with washing buffer (0.2% TritonX-100 and 0.05% Tween20 in PBS) and incubated with primary antibodies: anti-Ki67 antibody (Abcam, ab92742, 1:50), or anti-E-Cadherin antibody (Abcam, ab1416, 1:50) in 0.2% BSA in PBS at 4 °C overnight. After staining with the primary antibodies, samples were rinsed with washing buffer and incubated with secondary antibodies (1:400): Alexa Fluor 488 Goat anti-rabbit IgG (Invitrogen, A11008) or Alexa Fluor 488 Goat anti-mouse IgG (Invitrogen, A11001) in 0.2% BSA in PBS at 4 °C overnight. Organoids and tumoroids were rinsed with washing buffer and F-actin was stained with Phalloidin (Alexa Fluor™ 546 Phalloidin, Invitrogen, A22283.1:80) in PBS for 30 min at room temperature followed by a wash in PBS. Before imaging, nuclei were stained with DAPI (Sigma. MBD0015, 1:1000) for 10 min at room temperature and the residual DAPI was washed off with PBS. Organoids and tumoroids in PBS were transferred into a µ-Slide 8 Well slide (Ibidi, 80826) and images were captured using a Leica SP8 Confocal microscope with a 40x objective and Leica LAS X LS software.

### Whole-genome sequencing

A total of 26 tumoroids were sequenced on a DIPSEQ platform (BGI). Sequencing libraries were constructed according to the instructions of the MGIEasy FS DNA Library Prep Set (MGI, item No.: 1000006987), and 2× 100-bp paired-end sequencing was performed to yield data of estimated ≥30× coverage. Pre-processing, including removal of low-quality reads and adaptor sequences, was carried out using SOAPnuke (v2.0.7)^45^. High-quality reads were mapped and processed for downstream analysis using Sentieon Genomics software (version: sentieon-genomics-201911)^46^ which includes the following optimized steps: 1) aligned hg38 with BWA MEM^47^ with alt-aware mapping model; 2) sorted alignment reads by Samtools^48^; 3) marked duplicate reads by Picard (http://broadinstitute.github.io/picard/); 4) indel realignment and base quality score recalibration for alignment reads by GATK^49^; and 5) alignment QC by Picard. In the absence of a matched normal, putative somatic SNVs and INDELs were identified and filtered by Mutect2^50^ and FilterMutectCalls (GATK v4.0.10.1)^50^ in tumor-only mode; only “PASS” or “germline_risk” sites were kept. Putative somatic mutations were identified by filtering out SNVs and INDELs found with a 0.0001 or higher frequency in the Genome Aggregation Database^51^ and 0.0005 or higher frequency in the 1000 Genomes Project^52^ as well as polymorphisms identified in five reference normal samples sequenced on the same platform. Putative somatic SNVs and INDELs were annotated with the Personal Cancer Genome Reporter (PCGR) (v0.9.0)^53^. Putative CNVs for each tumoroid were identified using the CNVkit (v0.9.7) with a flat reference as control (sequence-accessible coordinates, 5 kb; average bin size, 1 kb; CBS segmentation algorithm, CBS)^54^. Whole-genome sequencing data are deposited in the CNGB Sequence Archive (CNSA) under project accession number CNP0001424 and the NCBI Sequence Read Archive (SRA) database with project accession number PRJNA682490.

The Cancer Genome Atlas (TCGA)-derived exome captured sequencing data for somatic SNVs and INDELs were retrieved for 224 colorectal cancer samples as described previously^9,55^. Segmented DNA copy-number data were retrieved via the Genomic Data Commons Data Portal (https://portal.gdc.cancer.gov/).

### Microsatellite instability analysis

Microsatellite instability (MSI) analysis to determine the DNA mismatch-repair (dMMR) status of tumoroids was performed for the microsatellite markers BAT25 and BAT26 using fluorescently labeled primers on a 3130xl Genetic Analyzer (Applied Biosystem) and data analyzed using GeneMapper Software v4.0 (Applied Biosystem). MSI-high (deficient dMMR) was diagnosed if instability was evident at one or more markers.

### Semiautomated drug assay

Tumoroids were digested with TrypLE Express enzyme at 37 °C for 15–20 min. Digestion was terminated in 1% BSA in DMEM/F12 and fragments were collected by centrifugation. Pellets were resuspended in culture medium, dissociated with a 26 G needle and filtered through a 40 μm cell strainer (Bio-Strategy, BDAA352340) to produce a single-cell suspension. Live cells were counted using a hemocytometer and trypan blue exclusion staining. Single cells were suspended in culture medium with 3% Matrigel, 250 U/ml penicillin-streptomycin, 100 µg/ml normocin and 10 μM Y27632 and seeded in 384-well optical plates (Thermo Fisher Scientific, NUN242764) (3000 cells in 60 µl/well) using a MANTIS® liquid-handling robot (Formulatrix), and established for 3 days before drug treatment.

For plate uniformity studies, 1 µM bortezomib (Selleck, S1013) was used as the positive control, and 0.5% (v/v) DMSO (Sigma, D2650) was used as a negative vehicle control. For the drug dose-response curves, regorafenib (a tyrosine kinase inhibitor, Selleck, S1178) was used in a nine point 4-fold titration series starting at 50 µM. All compound plates were prepared using a JANUS liquid-handling robot (PerkinElmer) and compounds were transferred into 384-well plates using an integrated PinTool addition accessory (PerkinElmer).

For drug sensitivity testing of standard-of-care chemotherapy agents for colorectal cancer, drugs were assayed in duplicate in a nine point, 4-fold dilution series with starting concentrations of 50 µM for 5-fluorouracil, 75 µM for oxaliplatin, 0.5 µM for SN-38, 50 µM for regorafenib and 50 µM for TAS-102. DMSO 0.5% (v/v) and 1 µM bortezomib served as negative and positive controls, respectively.

Tumoroid cultures were imaged every 24 h for 7 days by bright-field microscopy on an automated Nikon Eclipse Ti2 Inverted Microscope System with an integrated tissue culture incubator. 384-well images were captured with a 4× objective and NIS-Elements AR software (version 5.21.03, 64-bit) with a 25 µm Z-stack over a range of 500 μm. On day 7, 20 µl CellTiter-Glo 3D reagent (Promega, G9683) was added using the MANTIS® liquid-handling robot, plates were shaken at 1000 rpm on a IKA® MS 3 digital shaker for 30 mins at room temperature and luminescence measured using an EnVision plate reader (PerkinElmer).

### Computational image analysis

For image analysis, flattening of Z-stack bright-field images, removal of background debris and selection of organoids were performed using ImageJ software (NIH Image)^56^. The projection of z-stack images onto a single in-focus image was carried out using the Extended Depth of Field (EDF) plugin (http://bigwww.epfl.ch/demo/edf/) in “easy mode,” with the parameter quality = ‘1’ and topology = ‘1.’ Processed images were exported as high-quality tiff files. Background debris removal was performed by with a series of steps including Subtract Background, Enhance Local Contrast (CLAHE), set AutoThreshold, set Black Background, and Convert to Mask. Tumoroid selection and feature extraction were achieved using sequential Analyze Particles, Invert LUT, Fill Holes, Watershed, Set Measurements, and Analyze Particles steps. Features of selected tumoroids were exported as text files for determination of mean tumoroid size.

### Dose-response quantitation

Growth rate-adjusted organoid viability (GR) values were calculated for imaging data based on day 0 negative control, day 7 negative/positive control, and day 7 drug-treated wells as described by Hafner et al. using Eq. (1)^33^:

1 $${{{{{\rm{GR}}}}}}({{{{{\rm{c}}}}}})={2}^{({\log }_{2}({{{{{\rm{x}}}}}}({{{{{\rm{c}}}}}})/{{{{{\rm{x}}}}}}_{0})/{\log }_{2}({{{{{{\rm{x}}}}}}}_{{{{{{\rm{ctrl}}}}}}}/{{{{{{\rm{x}}}}}}}_{0}))}-1.$$

Where *x*(*c*) is the mean tumoroid size for treated wells at the end point, *x*_Ctrl_ is the mean tumoroid area of DMSO controls at the end point and *x*_0_ is the mean tumoroid area of control wells at time 0. Mean size of tumoroid debris remaining in positive (killing) control wells was subtracted from these values. Growth rate-adjusted response curves were fitted using a 4-parameter log-logistic function in the “DRC” package. GR_50_ (concentration of drug that reduces cell proliferation rate by one-half), GR_max_ (GR value at maximum dose) and GR_aoc_ (area over curve) were calculated as described by Hafner et al.^33^

For comparison of uniformity of regorafenib dose-response curves between the imaging and CellTitre-Glo 3D data, response curves were fitted using a four-parameter log-logistic function to percent viability data.

### Evaluation of assay quality

Tumoroid assays in 384-well plates were examined for quality using Eqs. (2)–(5):

2 $${{{{{\rm{Coefficient}}}}}}\,{{{{{\rm{of}}}}}}\,{{{{{\rm{variation}}}}}}:{{{{{\rm{CV}}}}}}={{{{{\rm{s}}}}}}.{{{{{\rm{d}}}}}}./{{{{{\rm{mean}}}}}}\times 100 \%$$

3 $${{{{{\rm{Z}}}}}}^{\prime} {{{{{\rm{factor}}}}}}:\,{{{{{\rm{Z}}}}}}^{\prime} =1-3\times ({{{{{\rm{s}}}}}}.{{{{{\rm{d}}}}}}.({{{{{{\rm{C}}}}}}}_{{{{{{\rm{pos}}}}}}})+{{{{{\rm{s}}}}}}.{{{{{\rm{d}}}}}}.({{{{{{\rm{C}}}}}}}_{{{{{{\rm{neg}}}}}}}))/{{{{{\rm{abs}}}}}}({{{{{\rm{mean}}}}}}({{{{{{\rm{C}}}}}}}_{{{{{{\rm{pos}}}}}}})-{{{{{\rm{mean}}}}}}({{{{{{\rm{C}}}}}}}_{{{{{{\rm{neg}}}}}}}))$$

4 $${{{{{\rm{Robust}}}}}}\,{{{{{\rm{Z}}}}}}^{\prime} {{{{{\rm{factor}}}}}}:{{{{{\rm{RZ}}}}}}^{\prime} =1-3\times ({{{{{\rm{m}}}}}}.{{{{{\rm{a}}}}}}.{{{{{\rm{d}}}}}}.({{{{{{\rm{C}}}}}}}_{{{{{{\rm{pos}}}}}}})+{{{{{\rm{m}}}}}}.{{{{{\rm{a}}}}}}.{{{{{\rm{d}}}}}}.({{{{{{\rm{C}}}}}}}_{{{{{{\rm{neg}}}}}}}))/{{{{{\rm{abs}}}}}}({{{{{\rm{median}}}}}}({{{{{{\rm{C}}}}}}}_{{{{{{\rm{pos}}}}}}})-{{{{{\rm{median}}}}}}({{{{{{\rm{C}}}}}}}_{{{{{{\rm{neg}}}}}}}))$$

5 $${{{{{\rm{Signal}}}}}}\,{{{{{\rm{window}}}}}}:{{{{{\rm{SW}}}}}}=({{{{{\rm{abs}}}}}}({{{{{\rm{mean}}}}}}({{{{{{\rm{C}}}}}}}_{{{{{{\rm{pos}}}}}}})-{{{{{\rm{mean}}}}}}({{{{{{\rm{C}}}}}}}_{{{{{{\rm{neg}}}}}}}))-3\times ({{{{{\rm{s}}}}}}.{{{{{\rm{d}}}}}}.({{{{{{\rm{C}}}}}}}_{{{{{{\rm{pos}}}}}}})+{{{{{\rm{s}}}}}}.{{{{{\rm{d}}}}}}.({{{{{{\rm{C}}}}}}}_{{{{{{\rm{neg}}}}}}})))/{{{{{\rm{s}}}}}}.{{{{{\rm{d}}}}}}.({{{{{{\rm{C}}}}}}}_{{{{{{\rm{pos}}}}}}})$$

Assays meeting criteria of CV < 20%, standard Z’ or robust Z’ >0.4 and SW > 2 were defined as acceptable^27^.

### Statistics and Reproducibility

Statistical analyses were performed using the statistical computing software R (*R* Development Core Team, 2011). Differences between the groups were assessed using the Fisher’s exact test for categorical variables and the Student’s *t* test for continuous variables. Correlations were assessed using the t test and Pearson’s correlation coefficient. Error bars represent s.d. or s.e.m., as specified. All statistical analyses were two-sided and considered significant if *p* < 0.050.

### Reporting summary

Further information on research design is available in the Nature Research Reporting Summary linked to this article.

## Supplementary information

Supplementary Information

Description of Supplementary Files

Supplementary Data 1

Reporting Summary

## Data Availability

Whole-genome sequencing data have been deposited into the CNGB Nucleotide Sequence Archive (CNSA) of CNGBdb with project accession number CNP0001424 (https://db.cngb.org/cnsa/)^57,58^ and the NCBI Sequence Read Archive (SRA) database with project accession number PRJNA682490. Source data are provided in Supplementary Data 1.
