# Supplementary material for: Low-viscosity matrix suspension culture enables scalable analysis of patient-derived organoids and tumoroids from the large intestine
Source: Commun Biol. 2021 Sep 13;4:1067. doi: 10.1038/s42003-021-02607-y (PMC8438070; doi:10.1038/s42003-021-02607-y)
Supplement: Supplementary file 1 — Supplementary Information [file 42003_2021_2607_MOESM1_ESM.pdf]

SUPPLEMENTARY FIGURES

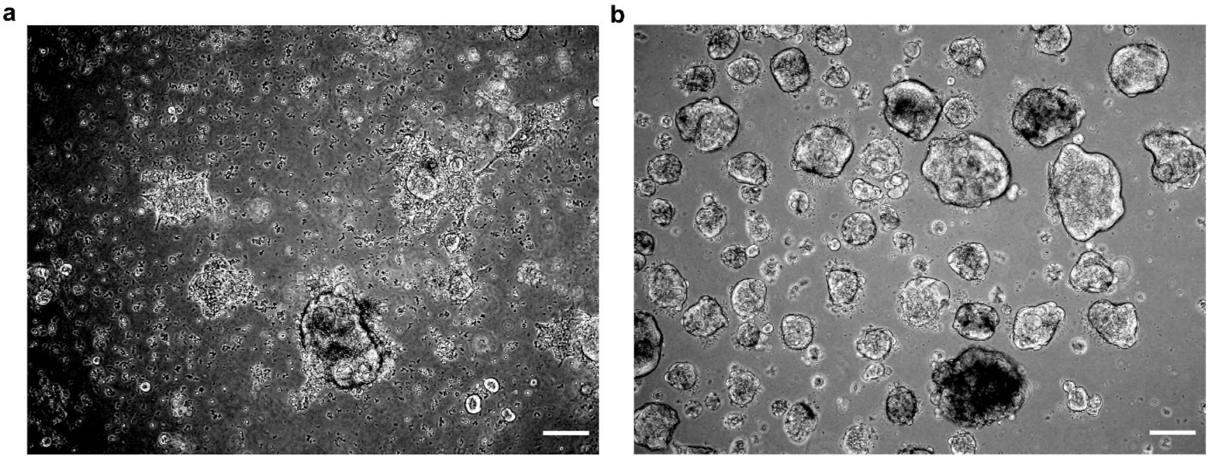

Supplementary Fig 1. Colorectal tumoroids grown in low-viscosity matrix suspension culture without and with repeated agitation. Representative images of tumoroids grown (**a**) without and (**b**) with gentle agitation by pipetting every 2-3 days; scale bars, 100 μm.

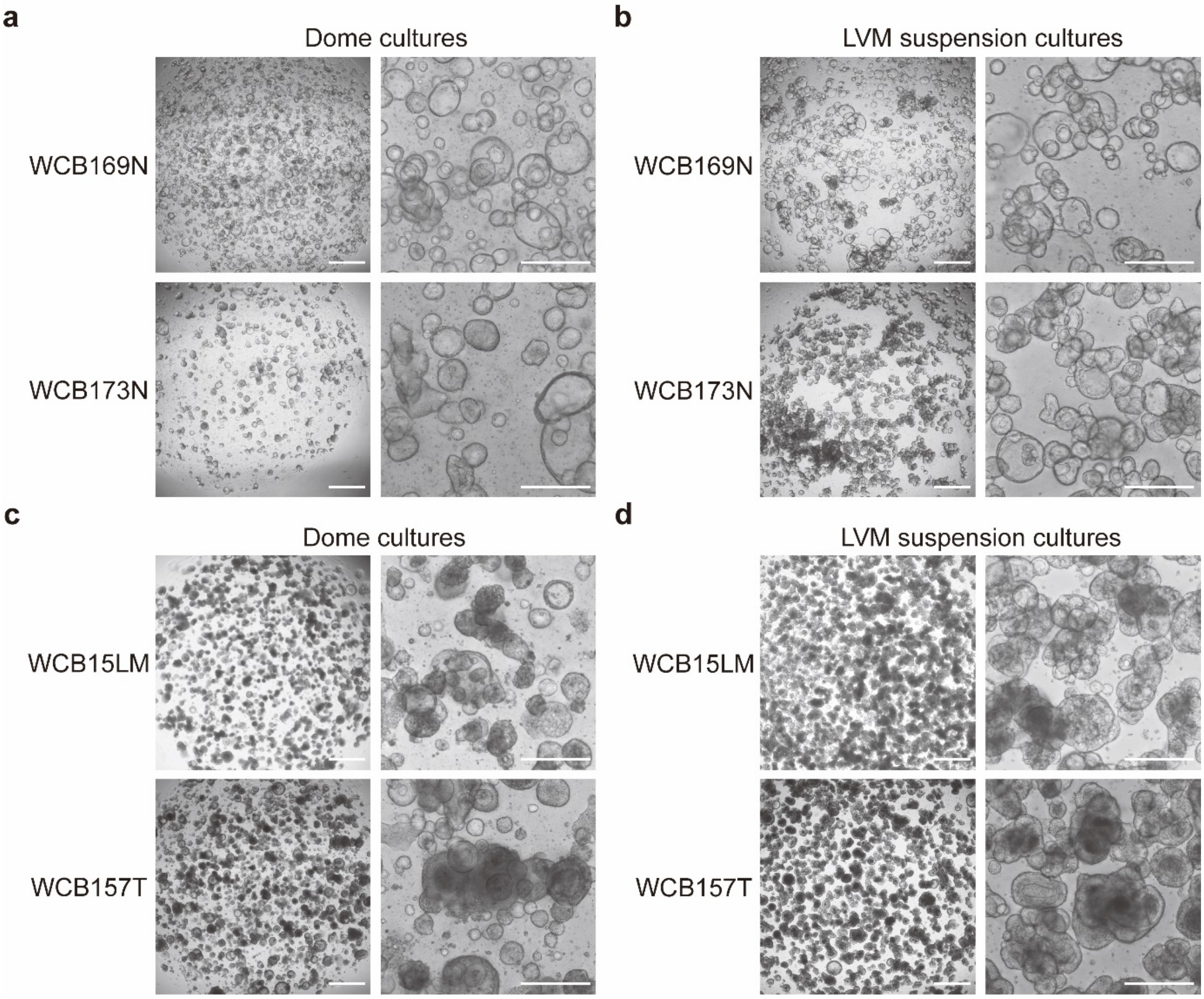

Supplementary Fig. 2. Low-viscosity matrix suspension culture for propagation of organoids and tumoroids from the human large intestine. a-b, Representative images of two independent normal colorectal organoids grown for 14 days in (**a**) Matrigel dome culture and (**b**) LVM suspension culture. **c-d**, Representative images of two independent colorectal tumoroids grown for 14 days in (**c**) Matrigel dome culture and (**d**) LVM suspension culture; low-magnification images, scale bars, 500 μm; high-magnification images, scale bars, 200 μm. N, normal; T, tumor; LM, liver metastasis.

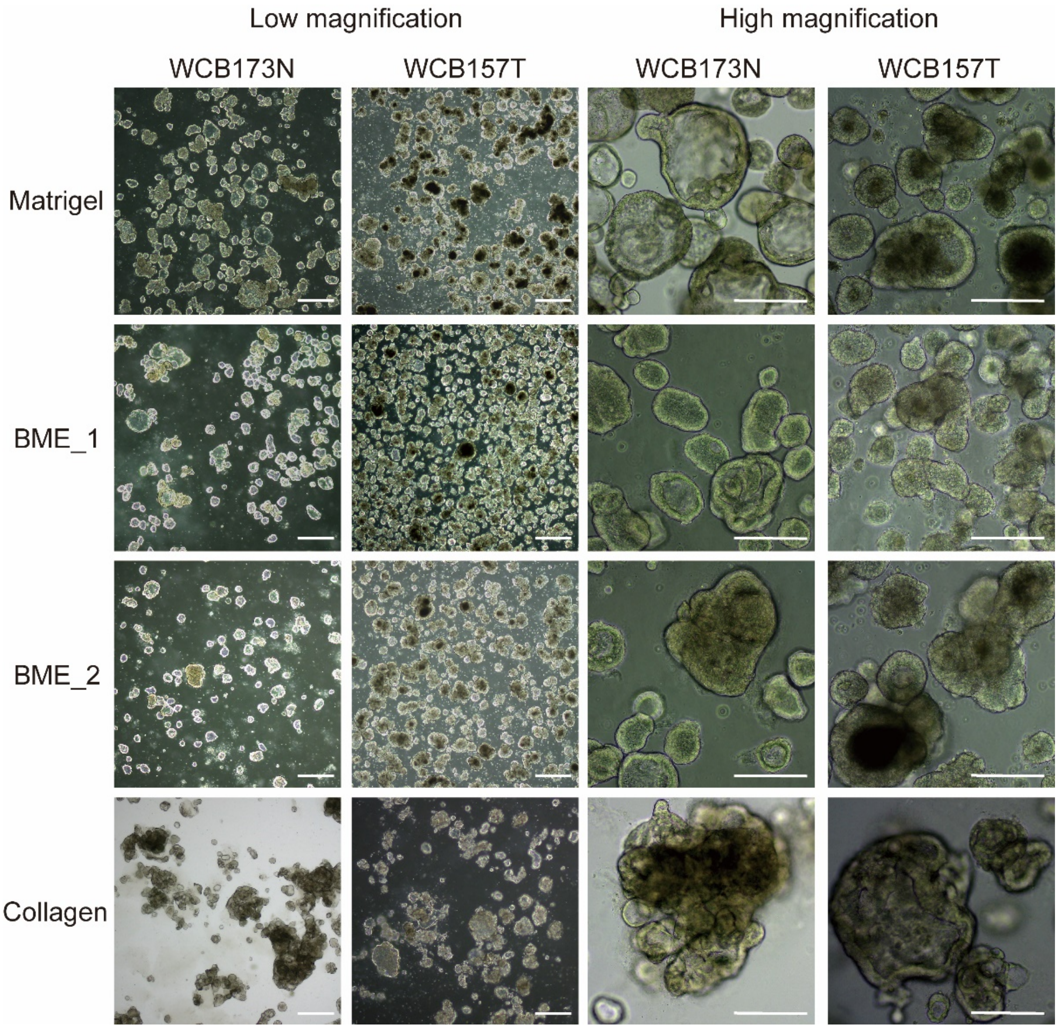

Supplementary Fig. 3. Low-viscosity matrix suspension cultures of both colorectal organoids and tumoroids using different types of support matrices. Matched organoids and tumoroids from a representative patient were grown for 14 days as suspension cultures in 5% Matrigel, BME-1, BME-2 or collagen type 1A; low-magnification images, scale bars, 500 µm; high-magnification images, scale bars, 200 µm. N, normal; T, tumor.

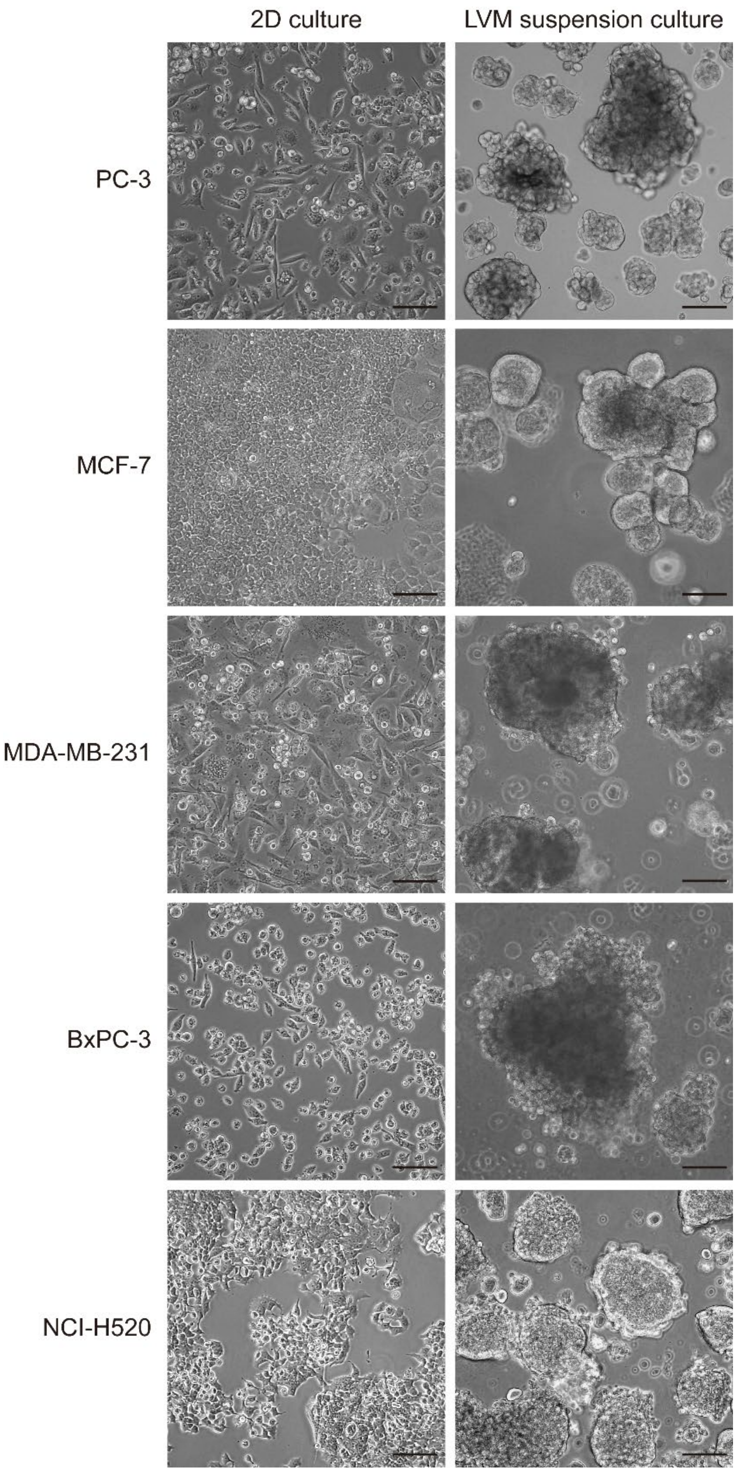

Supplementary Fig. 4. Low-viscosity suspension culture conditions support three-dimensional growth of human cancer cell lines. Representative images from human cancer cell lines from prostate (PC-3), breast (MCF-7, MDA-MB-231), pancreas (BxPC-3) and lung (NCI-H520) grown for 7 to 10 days in classic 2-D conditions and LVM suspension conditions; scale bars, 100 μm.

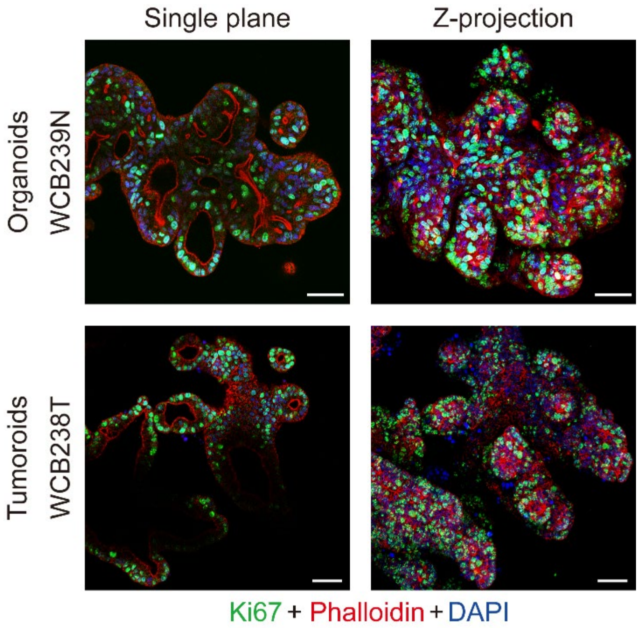

Supplementary Fig. 5. Morphology of colorectal organoids and tumoroids grown as dome cultures. Representative immunofluorescence microscopy images of organoids and tumoroids stained with Ki67 antibodies (green), phalloidin (for actin, red) and DAPI (blue); scale bars, 50 μm. N, normal; T, tumor.

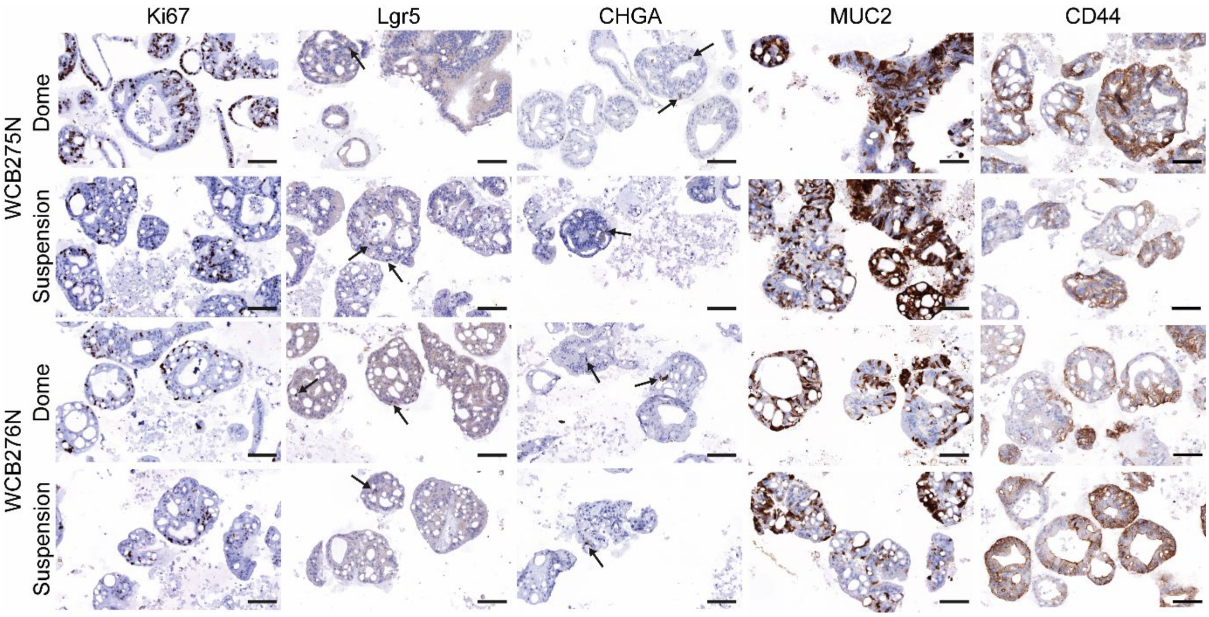

Supplementary Fig. 6. Marker expression for colorectal organoids grown as low-viscosity suspension or dome cultures. Immunohistochemistry for organoids from two patients stained for Lgr5, CD44, Ki67, MUC2 and CHGA; scale bars, 100 μm. N, normal.

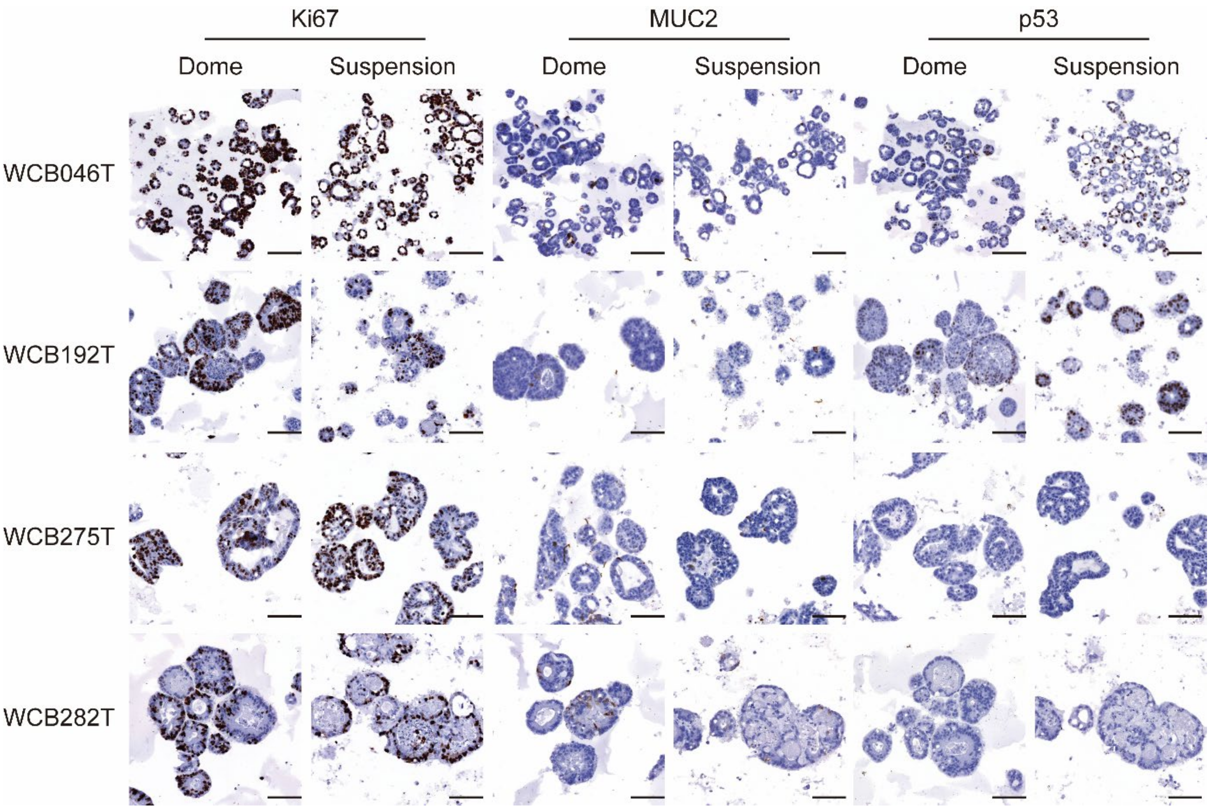

Supplementary Fig. 7. Immunohistochemistry for tumoroids from four patients stained for Ki67, MUC2 and p53; scale bars, 100 μm. T, tumor.

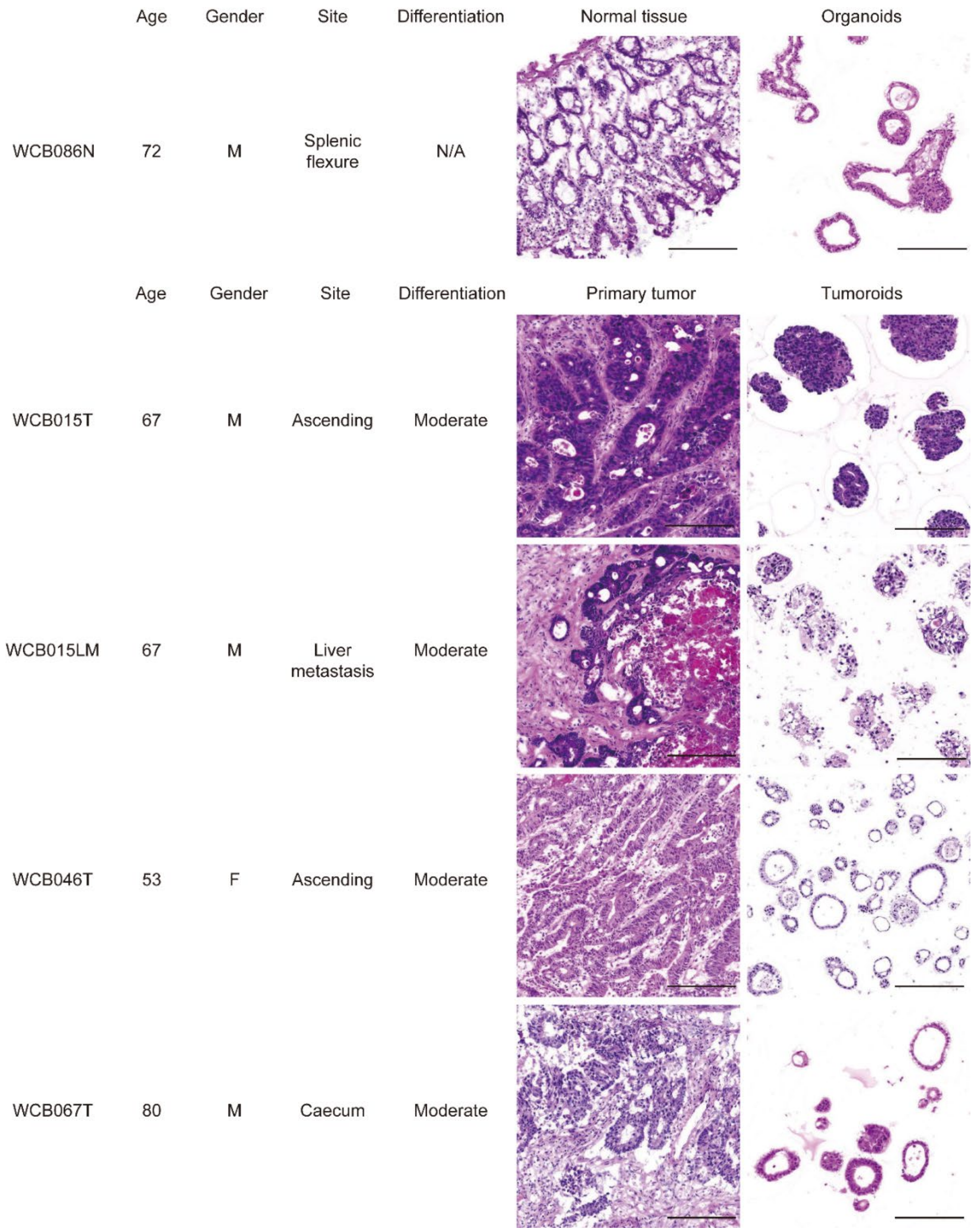

Supplementary Fig. 8. Colorectal organoids and tumoroids grown in low-viscosity matrix suspension culture maintain histopathological similarities as compared to the original primary tissues. Representative hematoxylin and eosin stained sections of matched normal tissue and organoids or matched tumor tissue and tumoroids from 5 patients; scale bars, 200 μm. N, normal; T, tumor; LM, liver metastasis.

	Age	Gender	Site	Differentiation
WCB079T	67	F	Ascending	Well
WCB088T	70	M	Sigmoid	Moderate
WCB143T	67	M	Sigmoid	Moderate
WCB181T	62	M	Sigmoid	Moderate
WCB184T	72	M	Sigmoid	Well
WCB189T	71	M	Caecum	Moderate

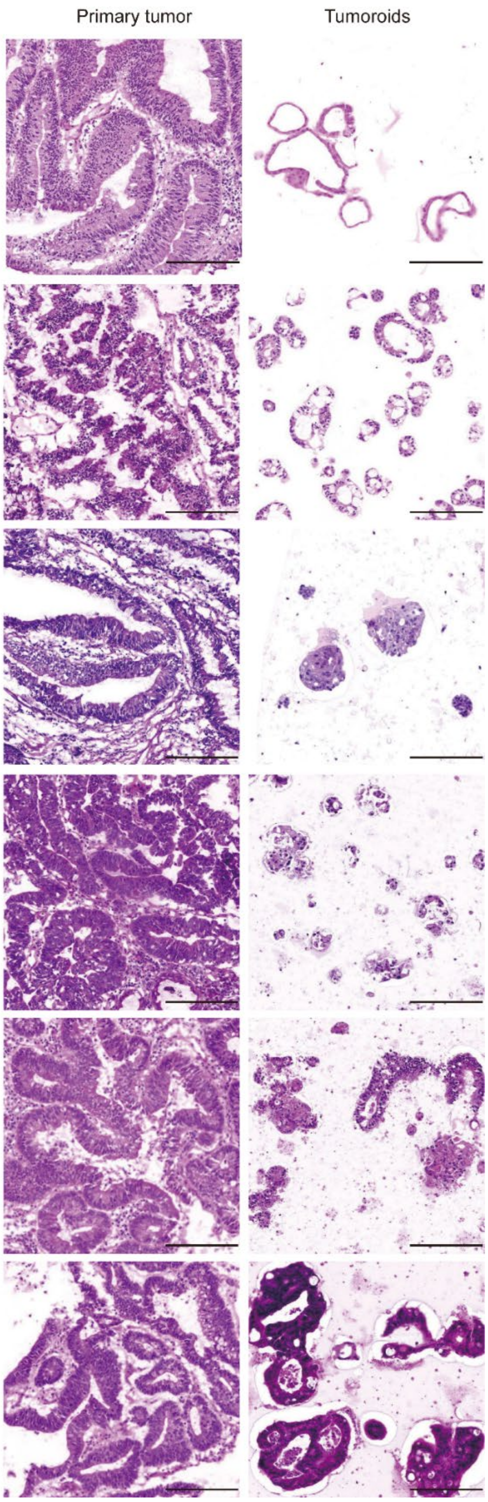

Supplementary Fig. 9. Colorectal organoids and tumoroids grown in low-viscosity matrix suspension culture maintain histopathological similarities as compared to the original primary tissues. Representative hematoxylin and eosin stained sections of matched tumor tissue and tumoroids from 6 patients; scale bars, 200 μm. N, normal; T, tumor; LM, liver metastasis.

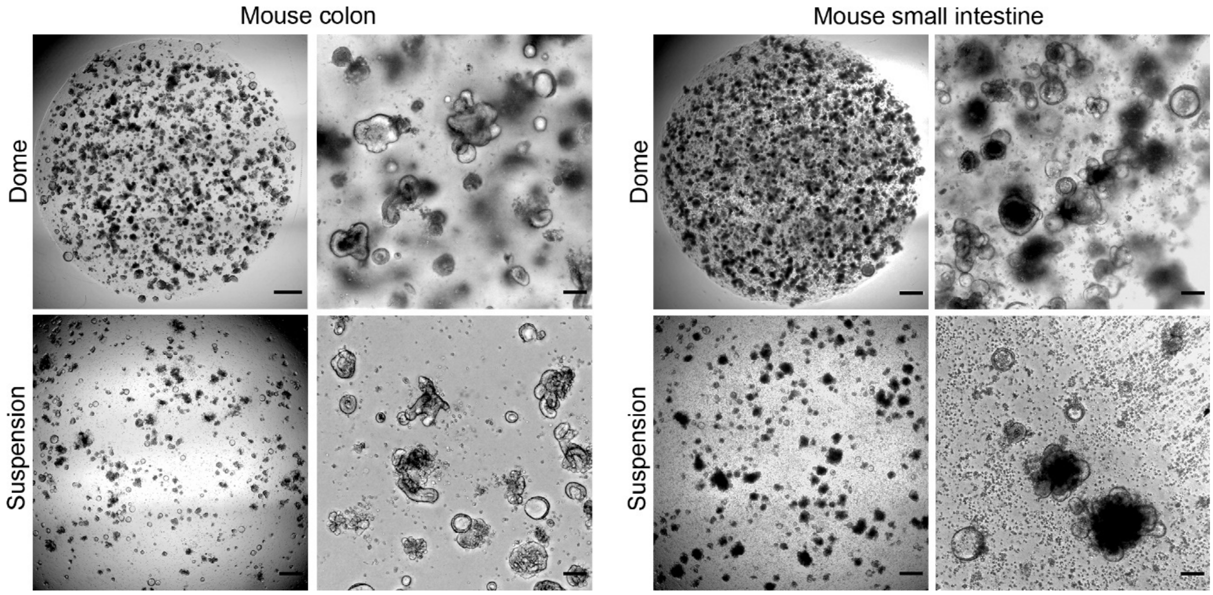

Supplementary Fig. 10. Low-viscosity suspension culture conditions support the growth of mouse intestinal organoids. Representative images of colonic organoids and small intestinal organoids established in either LVM suspension or dome culture; low magnification scale bars, 500 µm; high magnification scale bars, 100 µm.

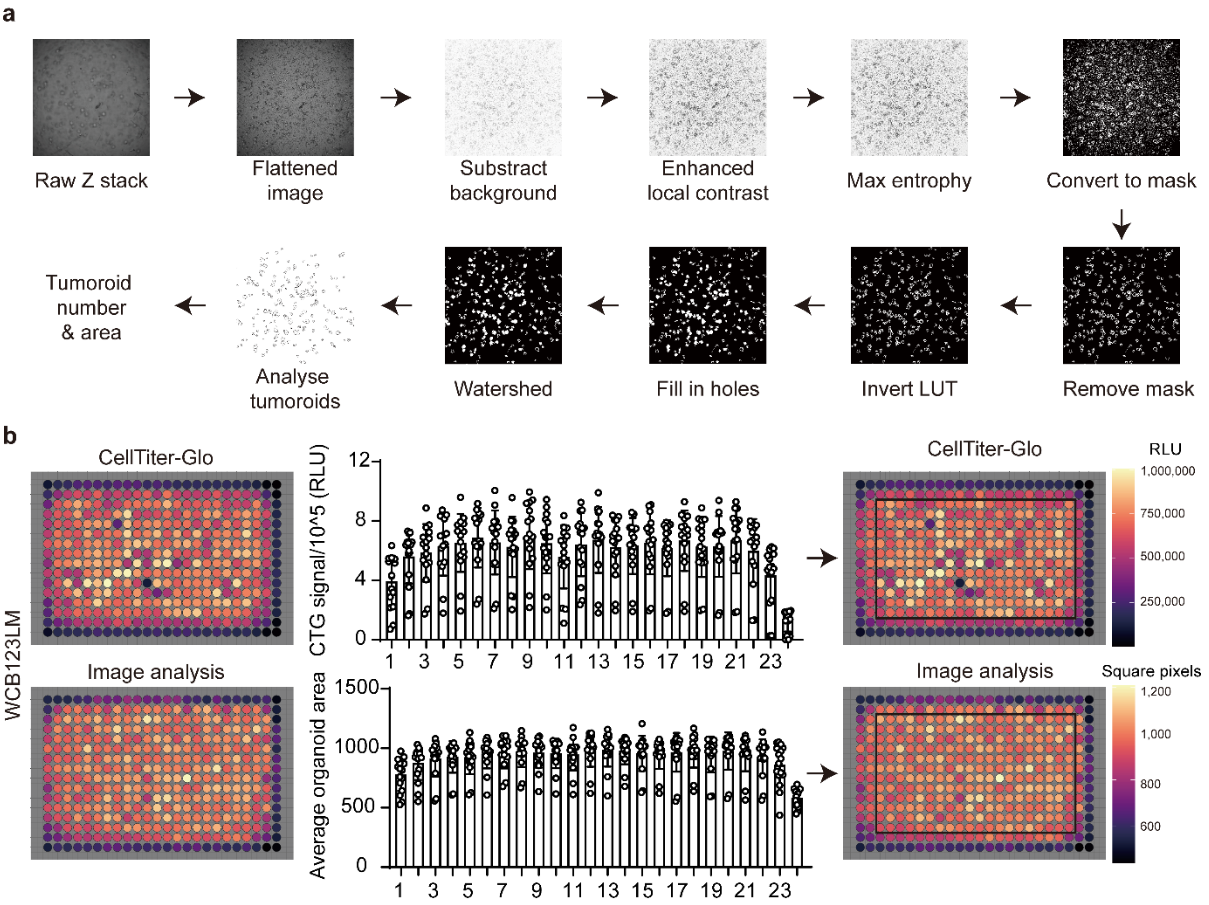

Supplementary Fig. 11. Low-viscosity matrix suspension culture of intestinal tumoroids in 384-well format. a, Overview of the image processing pipeline for determination of tumoroid sizes, including flattening of bright-field z-stack images, removal of background and identification of organoids. **b**, Assessment of plate edge effects for vehicle treated tumoroids after 10 days in culture. Mean tumoroid size and CellTitreGlo 3D luminescence signals were used for generating plate heatmaps; areas used for drug screen assays are marked with black boxes. Data are plotted as for each column as mean ± s.d. LM, liver metastasis.

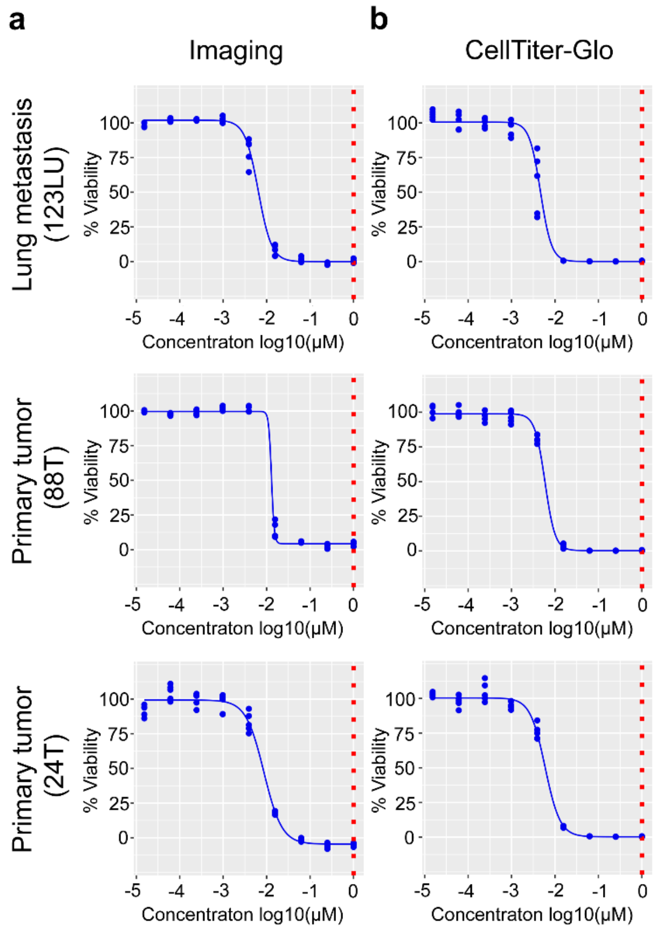

Supplementary Fig. 12. Inhibition of colorectal tumoroid growth using the proteasome inhibitor bortezomib. a-b, Three independent tumoroids were assayed in five replicate drug titrations and four-parameter logistic regression was used to fit drug dose-response curves to percentage viability values determined from (**a**) mean tumoroid size measurements by image analysis and (**b**) luminescence measurements by CellTitreGlo 3D assays. The bortezomib concentration of 1μm was selected as positive (killing) control, indicated by the dashed red line.

SUPPLEMENTARY TABLES

Supplementary Table 1. Propagation rates for organoids and tumoroids derived from 62 normal colorectal and 54 cancer tissues grown in LVM suspension or Matrigel dome culture. Statistical significance was attributed to values of $p<0.05$ as determined by the Fisher's exact test.

**Propagation**		**Dome culture**				**LVM suspension culture**				**Dome vs Suspension**
**Type**	**Characteristic**	**Yes**	**No**	**Success rate (%)**	**P value**	**Yes**	**No**	**Success rate (%)**	**P value**	**P value**
**Organoid**	**Site**									
	Right	16	2	88.9	0.207	13	2	86.7	1.000	1.000
	Left/Rectum	21	0	100.0		7	1	87.5		0.276
**Tumoroid**	**Site**									
	Right	10	1	90.9	1.000	7	4	63.6	0.667	0.311
	Left/Rectum	9	2	81.8		9	3	75.0		1.000
	**Stage**									
	I-III	19	3	86.4	1.000	14	4	77.8	0.277	0.680
	IV (incl. metastasis)	3	0	100.0		8	0	100.0		1.000
	**Differentiation**									
	Well/Moderate	16	1	94.1	0.352	15	6	71.4	0.318	0.104
	Poor	3	1	75.0		0	1	0		0.400

Supplementary Table 2. Propagation times for organoids and tumoroids derived from 62 normal colorectal and 54 cancer tissues grown in LVM suspension or Matrigel dome culture. Statistical significance was attributed to values of $p<0.05$ as determined by the Student's t test.

Propagation		Dome culture				LVM suspension culture				Dome vs Suspension
**Type**	**Characteristic**	**N**	**Mean (days)**	**S.D.**	**P value**	**N**	**Mean (days)**	**S.D.**	**P value**	**P value**
**Organoid**	**Site**									
	Right	16	20.2	11.7	0.949	13	25.5	10.5	0.181	0.233
	Left/Rectum	21	20.4	10.2		7	18.7	8.4		0.702
**Tumoroid**	**Site**									
	Right	10	23.5	10.7	0.626	7	21.7	9.0	0.732	0.739
	Left/Rectum	9	20.8	11.9		9	23.9	13.2		0.628
	**Stage**									
	I-III	19	22.2	11.4	0.450	14	22.6	11.5	0.534	0.918
	IV (incl. metastasis)	3	28.7	19.8		8	25.8	8.6		0.765
	**Differentiation**									
	Well/Moderate	16	20.5	10.6	0.145	15	23.9	11.3	N/A	0.407
	Poor	3	31.3	10.9		0	N/A	N/A		N/A

Supplementary Table 3. Establishment rates for organoids and tumoroids derived from 122 normal colorectal tissues and 91 cancer tissues grown in LVM suspension or Matrigel donut culture. Statistical significance was attributed to values of p<0.05 as determined by the Fisher's exact test. *p<0.05

Establishment		Donut culture				LVM suspension culture				Donut vs Suspension
**Type**	**Characteristic**	**Yes**	**No**	**Success rate (%)**	**P value**	**Yes**	**No**	**Success rate (%)**	**P value**	**P value**
**Organoid**	**Site**									
	Right	44	6	88.0	0.506	15	1	93.8	1.000	1.000
	Left/Rectum	38	3	92.7		14	1	93.3		1.000
**Tumoroid**	**Site**									
	Right	22	17	56.4	0.006*	5	1	83.3	1.000	0.377
	Left/Rectum	24	3	88.9		6	2	75.0		0.568
	**Stage**									
	I-III	43	19	69.4	0.330	10	3	76.9	1.000	0.744
	IV (incl. metastasis)	11	2	84.6		2	1	66.7		0.489
	**Differentiation**									
	Well/Moderate	40	16	71.4	0.440	10	3	76.9	1.000	1.000
	Poor	5	4	55.6		1	0	100		1.000

Supplementary Table 4. Establishment times for organoids and tumoroids derived from 122 normal colorectal tissues and 91 cancer tissues grown in LVM suspension or Matrigel donut culture. Statistical significance was attributed to values of p<0.05 as determined by the Student's t test. *p<0.05

Establishment		Donut culture				LVM suspension culture				Donut vs Suspension
**Type**	**Characteristic**	**N**	**Mean (days)**	**S.D.**	**P value**	**N**	**Mean (days)**	**S.D.**	**P value**	**P value**
**Organoid**	**Site**									
	Right	44	6.2	3.2	0.059	15	18.6	12.8	0.270	<0.001*
	Left/Rectum	38	7.7	3.9		14	14.1	7.1		<0.001*
**Tumoroid**	**Site**									
	Right	22	14.6	7.8	0.871	5	23.4	17.0	0.646	0.106
	Left/Rectum	24	14.2	10.9		6	19.5	6.0		0.272
	**Stage**									
	I-III	43	14.6	9.7	0.452	10	22.8	12.0	0.101	0.030*
	IV (incl. metastasis)	11	12.1	9.7		2	6.0	0.0		0.432
	**Differentiation**									
	Well/Moderate	40	13.7	9.2	0.216	10	22.8	12.0	N/A	0.013*
	Poor	5	19.4	11.5		1	6.0	0.0		N/A

Supplementary Table 5. Performance metrics for plate uniformity for colorectal tumoroids assessed using image analysis. Plate uniformity in 384-well format was evaluated for three different tumoroids for maximum (Max) signals, minimum (Min) signals, and drug dose-dependent midpoint (Mid) signals based on organoid size measurements with the latter reported for each side of each plate (left and right). CV, coefficient of variation; S.D. standard deviation; SW, signal window; Z', Z' factor. N = 228 for Max (Max plates) and Min (Min plates). N = 12 for Max (Min plates), Min (Max plates), and Mid (Mid plates, left and right). N = 6 for Max (Mid plates left and right), Min (Mid plates, left and right).

**Imaging**									
**Sample ID**	**Plate**	**Type**	**Mean (size)**	**S.D.**	**CV**	**SW**	**Z' (robust Z')**	**Mean ED_{50} (µM)**	**Mean ED_{50} Fold Change**
WCB024T	Mid (Left)	Max	942	38	4.0	10.9	0.73 (0.82)	1.54	1.5
		Min	376	13	3.5				
		0.78 uM	699	69	9.8				
	Mid (Right)	Max	963	37	3.8	13.1	0.78 (0.73)	2.28	
		Min	353	8	2.2				
		0.78 uM	730	53	7.3				
	Max	Max	714	42	5.9	4.3	0.51 (0.55)		
		Min	361	15	4.1				
	Min	Max	809	39	4.8	4.7	0.48 (0.50)		
		Min	427	35	6.1				
WCB088T	Mid (Left)	Max	623	22	3.5	8.2	0.59 (0.60)	6.09	1.7
		Min	325	19	5.8				
		12.50 uM	395	17	4.4				
	Mid (Right)	Max	617	25	4.1	7.1	0.61 (0.80)	10.15	
		Min	326	13	4.1				
		12.50 uM	441	27	6.2				
	Max	Max	722	30	4.1	6.4	0.55 (0.60)		
		Min	378	22	5.8				
	Min	Max	736	31	4.3	6.1	0.54 (0.64)		
		Min	378	25	6.1				
WCB123LU	Mid (Left)	Max	934	45	4.8	7.5	0.55 (0.68)	1.23	1.3
		Min	315	49	15.4				
		0.78 uM	661	35	5.3				
	Mid (Right)	Max	900	17	1.9	29.0	0.82 (0.82)	1.56	
		Min	286	18	6.5				
		0.78 uM	673	32	4.7				
	Max	Max	1052	46	4.4	10.8	0.67 (0.75)		
		Min	303	37	12.3				
	Min	Max	898	41	4.5	8.8	0.61 (0.60)		
		Min	302	27	12.2				

Supplementary Table 6. Performance metrics for plate uniformity for colorectal tumoroids assessed using CellTitre-Glo 3D assays. Plate uniformity in 384-well format was evaluated for three different tumoroids for maximum (Max) signals, minimum (Min) signals, and drug dose-dependent midpoint (Mid) signals based on fluorescence measurements with the latter reported for each side of each plate (left and right). CV, coefficient of variation; S.D. standard deviation; SW, signal window; Z', Z' factor. N = 228 for Max (Max plates) and Min (Min plates). N = 12 for Max (Min plates), Min (Max plates), and Mid (Mid plates, left and right). N = 6 for Max (Mid plates left and right), Min (Mid plates, left and right).

**CellTitre-Glo 3D**									
**Sample ID**	**Plate**	**Type**	**Mean (intensity)**	**S.D.**	**CV**	**SW**	**Z' (robust Z')**	**Mean ED_{50} (µM)**	**Mean ED_{50} Fold Change**
WCB024T	Mid (Left)	Max	695975	100078	14.4	3.9	0.56 (0.71)	3.69	1.4
		Min	2323	1130	48.7				
		3.13 µM	350277	85107	24.3				
	Mid (Right)	Max	863768	123802	14.3	3.9	0.56 (0.73)	5.15	
		Min	7667	2244	29.3				
		3.13 µM	424756	82633	19.5				
	Max	Max	323298	47655	14.7	3.7	0.55 (0.51)		
		Min	2730	376	13.8				
	Min	Max	860127	169492	19.7	2.1	0.41 (0.40)		
		Min	860127	169492	37.0				
WCB088T	Mid (Left)	Max	1408517	256294	18.2	2.5	0.45 (0.35)	1.98	1.7
		Min	1908	1468	76.9				
		3.13 µM	544057	159171	29.3				
	Mid (Right)	Max	1516397	124227	8.2	9.0	0.75 (0.76)	3.41	
		Min	13263	3172	23.9				
		3.13 µM	873049	183175	21.0				
	Max	Max	1921444	341406	17.8	2.6	0.46 (0.44)		
		Min	13318	2439	18.3				
	Min	Max	1797946	320976	17.9	2.6	0.46 (0.39)		
		Min	458	84	29.2				
WCB123LU	Mid (Left)	Max	735969	161089	21.9	1.5	0.34 (0.71)	1.01	1.1
		Min	1040	927	89.2				
		0.78 µM	434300	78260	18.0				
	Mid (Right)	Max	809775	115061	14.2	3.9	0.56 (0.60)	1.10	
		Min	8079	2339	29.0				
		0.78 µM	475470	125155	26.3				
	Max	Max	1253442	300265	24.0	1.1	0.27 (0.16)		
		Min	8687	1478	17.0				
	Min	Max	1299146	198646	15.3	3.5	0.54 (0.77)		
		Min	340	62	27.2				
