# Supplementary material for: Low-viscosity matrix suspension culture enables scalable analysis of patient-derived organoids and tumoroids from the large intestine
Source: Commun Biol. 2021 Sep 13;4:1067. doi: 10.1038/s42003-021-02607-y (PMC8438070; doi:10.1038/s42003-021-02607-y)
Supplement: Supplementary file 2 — Description of Supplementary Files [file 42003_2021_2607_MOESM2_ESM.pdf]

Description of Additional Supplementary Files

File name: Supplementary Data 1.

Description: Source data underlying Fig. 1a, Fig. 1b, Fig. 2b, Fig. 2f, Fig. 2g, Fig. 3c, Fig. 6b, Fig. 6f, Fig. 6g, Fig. 7c, Supplementary Fig. 11b.
