# Supplementary material for: Low-viscosity matrix suspension culture enables scalable analysis of patient-derived organoids and tumoroids from the large intestine
Source: Commun Biol. 2021 Sep 13;4:1067. doi: 10.1038/s42003-021-02607-y (PMC8438070; doi:10.1038/s42003-021-02607-y)
Supplement: Supplementary file 4 — Reporting Summary [file 42003_2021_2607_MOESM4_ESM.pdf]

nature research

Corresponding author(s): Oliver Sieber
Last updated by author(s): Aug 9, 2021

Reporting Summary

Nature Research wishes to improve the reproducibility of the work that we publish. This form provides structure for consistency and transparency in reporting. For further information on Nature Research policies, see our Editorial Policies and the Editorial Policy Checklist.

Statistics

For all statistical analyses, confirm that the following items are present in the figure legend, table legend, main text, or Methods section.

n/a	Confirmed	
☐	☒	The exact sample size (*n*) for each experimental group/condition, given as a discrete number and unit of measurement
☐	☒	A statement on whether measurements were taken from distinct samples or whether the same sample was measured repeatedly
☐	☒	The statistical test(s) used AND whether they are one- or two-sided<br>*Only common tests should be described solely by name; describe more complex techniques in the Methods section.*
☐	☒	A description of all covariates tested
☐	☒	A description of any assumptions or corrections, such as tests of normality and adjustment for multiple comparisons
☐	☒	A full description of the statistical parameters including central tendency (e.g. means) or other basic estimates (e.g. regression coefficient) AND variation (e.g. standard deviation) or associated estimates of uncertainty (e.g. confidence intervals)
☐	☒	For null hypothesis testing, the test statistic (e.g. F, t, r) with confidence intervals, effect sizes, degrees of freedom and P value noted<br>*Give P values as exact values whenever suitable.*
☒	☐	For Bayesian analysis, information on the choice of priors and Markov chain Monte Carlo settings
☒	☐	For hierarchical and complex designs, identification of the appropriate level for tests and full reporting of outcomes
☐	☒	Estimates of effect sizes (e.g. Cohen's d, Pearson's r), indicating how they were calculated

Our web collection on statistics for biologists contains articles on many of the points above.

Software and code

Policy information about availability of computer code

Data collection	CaseViewer Software (3DHISTECH Ltd.), Leica LAS X LS software, NIS-Elements AR software (version 5.21.03, 64-bit), NIS-Elements BR software (version 4.40.00, 64-bit), TCapture software (version 5.1.1.0)
Data analysis	SOAPnuke (v2.0.7), Sentieon Genomics software (version: sentieon-genomics-201911), BWA MEM, Samtools, GATK, Picard, Mutect2, Personal Cancer Genome Reporter (PCGR) (v0.9.0), CNVkit (v0.9.7), ImageJ software (NIH Image), statistical computing software R (R Development Core Team, 2011)

For manuscripts utilizing custom algorithms or software that are central to the research but not yet described in published literature, software must be made available to editors and reviewers. We strongly encourage code deposition in a community repository (e.g. GitHub). See the Nature Research guidelines for submitting code & software for further information.

Data

Policy information about availability of data

All manuscripts must include a data availability statement. This statement should provide the following information, where applicable:
- Accession codes, unique identifiers, or web links for publicly available datasets
- A list of figures that have associated raw data
- A description of any restrictions on data availability

Whole-genome sequencing data have been deposited into the CNGB Nucleotide Sequence Archive (CNSA) of CNGBdb with project accession number CNP0001424 (https://db.cngb.org/cnsa/).

Field-specific reporting

Please select the one below that is the best fit for your research. If you are not sure, read the appropriate sections before making your selection.

☒ Life sciences ☐ Behavioural & social sciences ☐ Ecological, evolutionary & environmental sciences

For a reference copy of the document with all sections, see nature.com/documents/nr-reporting-summary-flat.pdf

Life sciences study design

All studies must disclose on these points even when the disclosure is negative.

Sample size	Organoids were derived from colorectal normal and cancer tissues from consecutive patients. Previous studies have reported success rates of ~70-80% for intestinal organoids from resection specimens, and accordingly group sizes of >15 were considered sufficient for evaluation of alternative novel culture approaches in this report.
Data exclusions	No data were excluded from the analysis.
Replication	Experiments were repeated on at least two independent occasions with consistent results.
Randomization	Consecutive samples were randomly allocated to reach group sizes of >15.
Blinding	Blinding was not possible as the aim of the study was to compare different culture conditions.

Reporting for specific materials, systems and methods

We require information from authors about some types of materials, experimental systems and methods used in many studies. Here, indicate whether each material, system or method listed is relevant to your study. If you are not sure if a list item applies to your research, read the appropriate section before selecting a response.

Materials & experimental systems

n/a	Involved in the study	
☐	☒	Antibodies
☐	☒	Eukaryotic cell lines
☒	☐	Palaeontology and archaeology
☐	☒	Animals and other organisms
☐	☒	Human research participants
☒	☐	Clinical data
☒	☐	Dual use research of concern

Methods

n/a	Involved in the study	
☒	☐	ChIP-seq
☒	☐	Flow cytometry
☒	☐	MRI-based neuroimaging

Antibodies

Antibodies used	Anti-Ki67 antibody (Abcam, ab92742); anti-E-Cadherin antibody (Abcam, ab1416); Alexa Fluor 488 Goat anti-rabbit IgG (Invitrogen, A11008), Alexa Fluor 488 Goat anti-mouse IgG (Invitrogen, A11001), anti-Ki67 antibody (clone MIB-1, DAKO, M7240), anti-Lgr5 antibody (clone OTI2A2, Thermo Fisher Scientific, TA503316, anti-Chr-A (CHGA) antibody (clone C-12, Santa Cruz Biotechnology, sc-393941), anti-Mucin 2 antibody (clone CCP58, Santa Cruz Biotechnology, sc-7314), anti-HCAM (CD44) antibody (Santa Cruz Biotechnology, sc7297) and anti-p53 antibody (clone DO-1, Santa Cruz Biotechnology, sc-126).
Validation	All antibodies are widely used, validated commercial products.

Eukaryotic cell lines

Policy information about cell lines

Cell line source(s)	Human prostate cancer cell line PC-3 (ATCC, CRL-1435), breast cancer cell lines MCF7 (ATCC, HTB-22) and MDA-MB-231 (ATCC, HTB-26), pancreatic cancer cell line BxPC3 (ATCC, CRL-1687) and lung cancer cell line NCI-H520 (ATCC, HTB-182).
Authentication	All cell lines were authenticated by STR analysis using the GenePrint 10 System (Promega) at the Australian Genome Research Facility (AGRF).
Mycoplasma contamination	All cell lines and organoid cultures were tested and found to be negative for mycoplasma contamination.
Commonly misidentified lines (See ICLAC register)	N/A

Animals and other organisms

Policy information about studies involving animals; ARRIVE guidelines recommended for reporting animal research

Laboratory animals	Mus musculus, C57/B16, male, 8.5 weeks
Wild animals	N/A
Field-collected samples	N/A
Ethics oversight	Walter and Eliza Hall Institute of Medical Research, AEC 2018.038

Note that full information on the approval of the study protocol must also be provided in the manuscript.

Human research participants

Policy information about studies involving human research participants

Population characteristics	Tumor and adjacent normal tissue samples were obtained from a community-based series of consecutive patients with colorectal adenocarcinoma.
Recruitment	Patients were recruited at the Western Health Hospital Footscray, Eastern Health Hospital Box Hill, Northern Health Hospital Epping and Royal Melbourne Hospital Parkville in Australia between 2017 and 2020.
Ethics oversight	This study was conducted in accordance with the Declaration of Helsinki, the NHMRC Statement on Ethical Conduct in Human Research and Institutional Human Research Ethics approval (HREC 2016.249). All patients gave informed consent.

Note that full information on the approval of the study protocol must also be provided in the manuscript.
